# Beyond IHC-Vicariation duality: precision-stratified rTMS for post-stroke motor recovery

**DOI:** 10.3389/fneur.2026.1856547

**Published:** 2026-10-26

**Authors:** Dai Zhang, Chenyu Zhao, Yuewen Ma

**Affiliations:** 1 Department of Rehabilitation Medicine, The First Hospital of China Medical University, China Medical University, Shenyang, China; 2 Department of China Medical University, The Queen’s University of Belfast Joint College, School of Pharmacy, China Medical University, Shenyang, China; 3 School of Pharmacy, Queen’s University Belfast, Belfast, United Kingdom; 4 Department of Biochemistry, Yong Loo Lin School of Medicine, National University of Singapore, Singapore, Singapore

**Keywords:** neurorehabilitation strategy, post-stroke recovery, precision medicine, repetitive transcranial magnetic stimulation, translational medicine

## Abstract

Stroke is the leading cause of long-term disability in adults, and refractory upper extremity motor dysfunction severely compromises patients’ ability to live independently. Repetitive transcranial magnetic stimulation (rTMS), which promotes neuroplasticity by modulating cortical excitability, has emerged as a highly promising therapeutic approach. However, its clinical efficacy exhibits high heterogeneity, mainly because current therapeutic strategies are guided by two seemingly opposing neuroscientific models. The classic Interhemispheric Competition (IHC) model advocates for inhibiting the unaffected hemisphere, while the emerging Vicariation model proposes leveraging compensatory functions of the unaffected hemisphere. This review aims to reconcile this theoretical divergence from a dialectical perspective and propose a biomarker-based individualized stratified treatment framework. The article first systematically reviews the neurophysiological evidence for both models, demonstrating that they are not mutually exclusive but rather applicable to different stages of neurological injury severity. Subsequently, it focuses on elaborating how the structural reserve assessment system, centered on corticospinal tract (CST) integrity and motor evoked potential (MEP), serves as a decision-making fulcrum to guide the selection of rTMS strategies including inhibition, excitation or bilateral modulation. The article proposes an evidence-informed stratification framework and explores the potential synergistic effects of emerging techniques such as theta-burst stimulation (TBS) and peripheral-central combined stimulation. Through multimodal patient stratification, this framework aims to support more individualized and mechanistically informed application of rTMS in post-stroke motor rehabilitation.

## Graphical abstract

**Figure:**
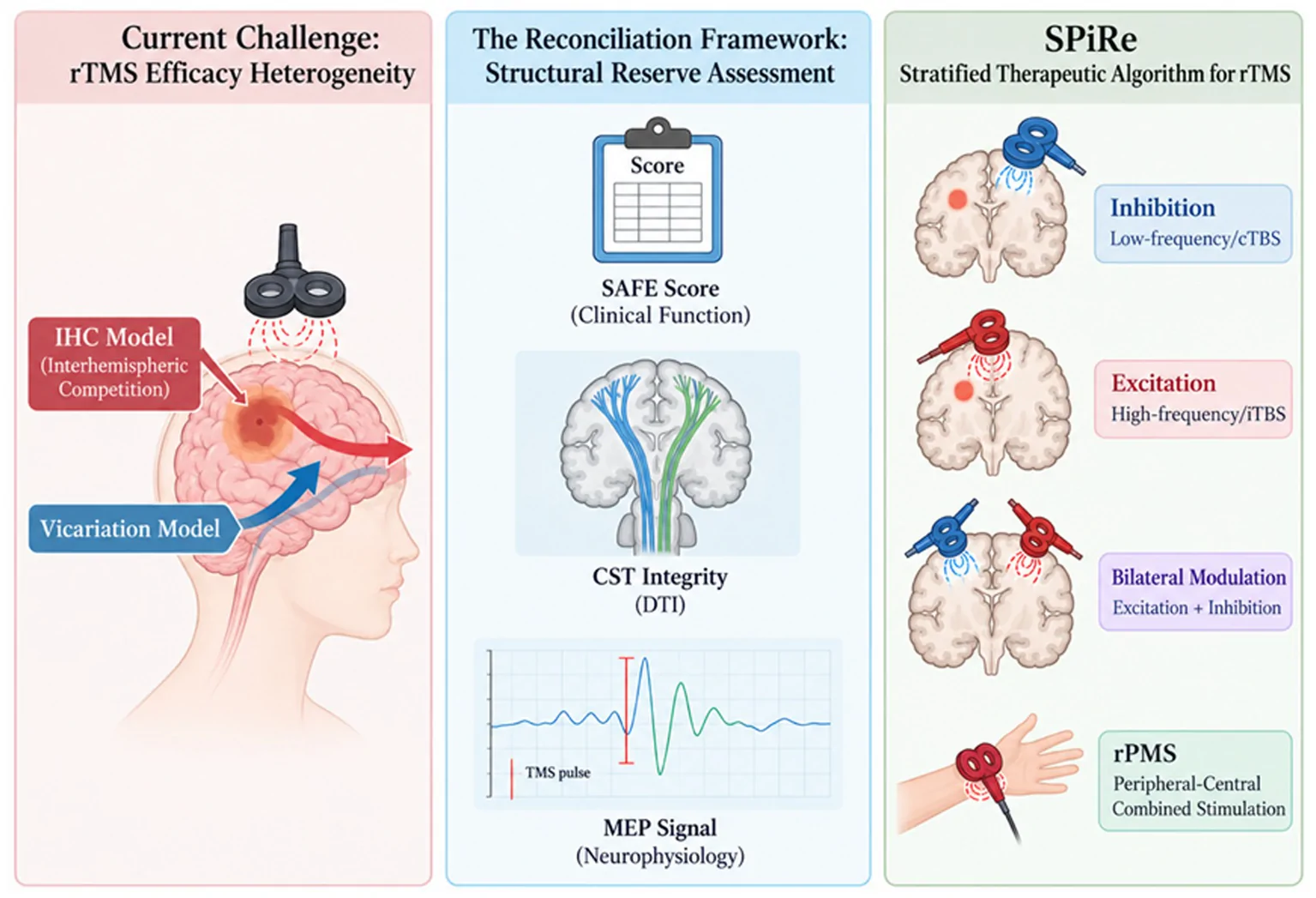

## Highlights

- Reconciled the theoretical conflict between Interhemispheric Competition and Vicariation models as severity-dependent mechanisms rather than opposing binaries.
- Conceptualized structural reserve, informed by CST integrity and MEP status, as a central decision-making fulcrum for biomarker-guided rTMS stratification.
- Proposed the SPiRe framework to support biomarker-informed selection among interhemispheric rebalancing and compensatory neuromodulation strategies.
- Highlighted that contralesional inhibition may be inappropriate in selected patients with severe CST disruption when contralesional networks contribute to compensatory motor output.
- Integrated emerging modalities like theta-burst and closed-loop stimulation into a precision medicine framework to optimize individualized recovery.

## 1 Introduction

Stroke represents more than an acute cerebrovascular incident; it serves as a catalyst for a prolonged process of central nervous system reorganization extending over months or even years (1, 2). While acute reperfusion therapies have significantly reduced mortality rates, epidemiological data indicate that more than 80% of survivors experience varying degrees of motor deficits six months post-onset (3–5). Loss of upper extremity motor control is notably intractable, as it undermines patients’ activities of daily living and functional independence in turn (6). Conventional physical and occupational therapies rely predominantly on repetitive peripheral sensorimotor input to drive central plasticity, a bottom-up strategy that frequently reaches a therapeutic plateau (7). In this context, repetitive transcranial magnetic stimulation (rTMS) has emerged as a transformative top-down intervention (8). Functioning on the principle of electromagnetic induction, rTMS modulates cortical excitability by inducing electric currents in the brain tissue (9). These currents provoke neuroplastic changes analogous to long-term potentiation (LTP) or long-term depression (LTD), offering a potent tool to overcome rehabilitation bottlenecks (10, 11).

Despite the theoretical promise of rTMS, its translational trajectory is hindered by substantial outcome heterogeneity (12). Indeed, the stark divide between responders and non-responders in clinical trials frequently consigns clinical practice to a trial-and-error loop (13). This inefficiency not only wastes the critical time window for recovery but also results in a significant drain on medical resources (14). The root of this uncertainty lies in the lack of consensus regarding optimal stimulation targets for individual patients, a confusion fundamentally driven by two conflicting neuroscientific models governing post-stroke recovery (15).

The field has traditionally been dominated by the IHC model (16). In a healthy brain, interhemispheric balance is maintained via transcallosal inhibition (TCI). The IHC model posits that stroke disrupts this equilibrium, as reduced output from the ipsilesional hemisphere leads to disinhibition of the contralesional side (17). This results in excessive, pathological TCI exerted upon the peri-lesional cortex (18, 19). Consequently, standard therapeutic protocols aim to restore balance by inhibiting the hyperactive contralesional hemisphere. However, this paradigm is increasingly challenged by the Vicariation model (20). Emerging evidence suggests that in patients with severe CST damage, the contralesional hemisphere does not act solely as a suppressor but may assume a supportive, compensatory role through uncrossed pathways or secondary motor areas (21). In such scenarios, applying inhibitory stimulation to the contralesional side based on the IHC model may inadvertently block this adaptive compensation and impede recovery (20).

A static, single-model approach is clearly insufficient to explain the dynamic complexity of recovery mechanisms, necessitating the establishment of an integrative framework. Therefore, this review aims to reconcile the theoretical divergence between the IHC and Vicariation models from a dialectical perspective, propelling rTMS application from a one-size-fits-all approach toward a precision-stratified paradigm. First, we delineate the technological rationale for selecting rTMS as the optimal modality for precision modulation. Second, we systematically review the conflicting neurophysiological evidence underpinning both the IHC and Vicariation models to expose the limitations of a binary theoretical approach. Third, we propose a dialectical synthesis wherein structural reserve, quantified via CST integrity and MEP, serves as the critical decision-making fulcrum for patient stratification. Finally, by integrating these elements, we propose the SPiRe framework as an evidence-informed approach for integrating multimodal biomarkers to support the selection of inhibitory, excitatory, or bilateral modulation strategies.

## 2 Rationale for rTMS selection

To circumvent the physiological plateau often encountered with conventional bottom-up rehabilitation, clinical focus has shifted toward top-down interventions capable of directly remodeling cortical networks (14). Within the expanding landscape of non-invasive brain stimulation, the selection of rTMS as the primary therapeutic modality is grounded in its superior neurophysiological potency compared to emerging alternatives. While techniques such as transcranial direct current stimulation (tDCS) are widely accessible, they function primarily through sub-threshold modulation. By altering resting membrane potentials without directly triggering action potentials, tDCS plays a largely adjunctive role and lacks the intensity required to drive robust plasticity in recalcitrant neural networks (22, 23). Similarly, emerging modalities like transcranial ultrasound stimulation (TUS) and temporal interference (TI) offer theoretical advantages regarding spatial resolution and depth targeting; however, these technologies remain in exploratory phases and currently lack the standardized safety protocols and established efficacy data necessary for widespread clinical translation (24, 25).

In contrast, rTMS utilizes electromagnetic induction to generate suprathreshold currents within the brain tissue, distinctively positioning it as the most mature and potent intervention for stroke rehabilitation (26). Unlike sub-threshold techniques, rTMS actively induces synchronized neuronal depolarization that leads to action potential generation (27). This mechanism effectively compels synaptic efficacy changes analogous to LTP or LTD, thereby providing the requisite physiological drive to override pathological network states (28). Consequently, rTMS stands as the established gold standard for inducing neuroplasticity. Yet, the possession of such a potent therapeutic tool does not guarantee clinical success. The efficacy of rTMS remains fundamentally constrained by a lack of consensus regarding the optimal direction of modulation, a challenge rooted in the conflicting theoretical models of post-stroke recovery that will be discussed in the following section (20, 29) (see Table 1).

**Table 1:** Comparative overview of non-invasive neuroregulatory technologies.

Modality	Mechanism of action	Physiological effect	Clinical maturity	Limitation relative to rTMS
rTMS	Electromagnetic induction	Directly triggers action potentials.	Established Gold Standard: Features mature hardware and standardized protocols.	Reference Standard: Currently evolving towards precise multimodal guidance.
tDCS	Weak direct current	Alters membrane potential thresholds without direct discharge.	Adjunctive: Primarily used for modulation rather than stimulation; simple hardware.	Insufficient Potency: Lacks the ability to directly induce neuronal firing; subject to skull attenuation.
TUS	Focused acoustic energy	Modulates activity via acoustic intensity.	Investigational: High spatial resolution but remains exploratory for clinical use.	Protocol Immaturity: Lacks the standardized safety and efficacy data established for rTMS.
TI	Interferential electric fields	Targets deep nuclei via intersecting high-frequency fields.	Emerging: Novel technique for deep-brain targeting.	Evidence Gap: Lacks the extensive clinical validation and safety profile accumulated by rTMS.

TI, temporal interference; tDCS, transcranial direct current stimulation; rTMS, repetitive transcranial magnetic stimulation; TUS, transcranial ultrasound stimulation. While emerging modalities offer theoretical advantages in depth, rTMS remains the superior choice for immediate clinical application due to its proven capacity for direct neuroplastic induction.

## 3 The theoretical duality: evidence and conflict

The advancement of non-invasive brain stimulation as a therapeutic modality for post-stroke motor rehabilitation has been propelled by a sophisticated yet increasingly contested understanding of interhemispheric interactions. At the heart of current clinical research lies a fundamental theoretical divergence. On one side stands the IHC model, a framework that has dominated the field for nearly three decades, positing that the healthy hemisphere exerts a maladaptive inhibitory force upon the lesioned hemisphere. On the other side is the Vicariation model, a perspective gaining ascendancy particularly in the context of severe injury, which argues that the healthy hemisphere serves as a critical compensatory reservoir (Figure 1). This chapter provides an exhaustive examination of these two opposing paradigms. We will dissect the neurophysiological mechanisms underpinning each model, evaluate the breadth of empirical evidence supporting their respective claims, and analyze the clinical paradoxes that emerge when these theories are applied indiscriminately to a heterogeneous patient population. By reconciling these seemingly contradictory frameworks, we lay the groundwork for a stratified, precision-medicine approach to rTMS.

**Figure 1:**
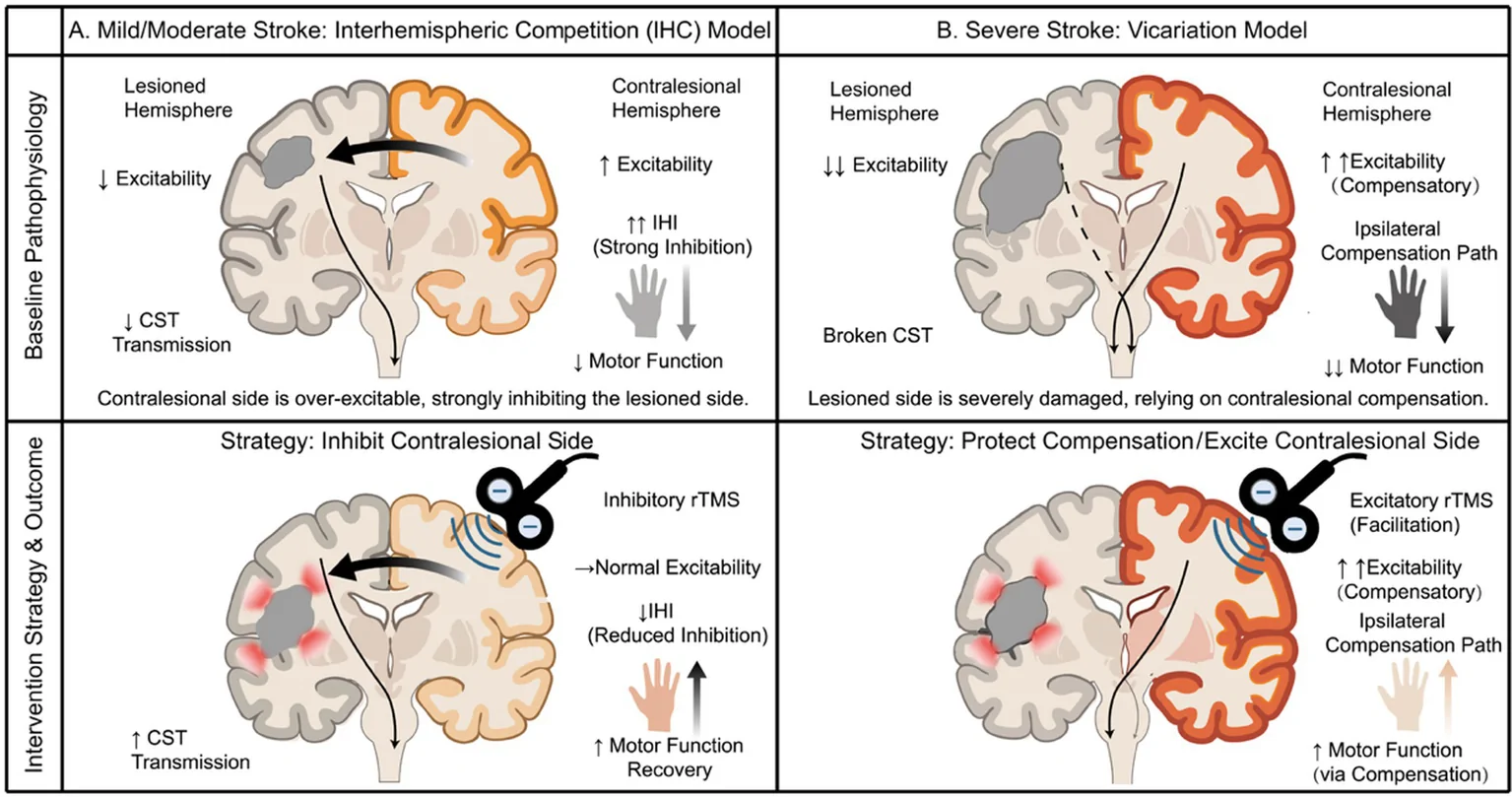
Schematic Representation of Pathophysiological Models Underlying Stroke Recovery and Tailored rTMS Intervention Strategies Based on Stroke Severity. This diagram contrasts two primary theoretical frameworks governing motor recovery and corresponding neuromodulation approaches. **(A)** Mild/Moderate Stroke: Interhemispheric Competition (IHC) Model. The top panel illustrates baseline pathophysiology where the contralesional hemisphere exhibits maladaptive high excitability, exerting strong interhemispheric inhibition (IHI) on the lesioned hemisphere and suppressing its residual corticospinal tract (CST) transmission. The bottom panel shows the therapeutic strategy of applying inhibitory rTMS to the contralesional hemisphere to reduce this inhibition, thereby normalizing excitability and promoting functional recovery of the lesioned hemisphere’s output. **(B)** Severe Stroke: Vicariation Model. The top panel illustrates a low-reserve state in which contralesional networks may contribute increasingly to residual motor output through ipsilateral or alternative descending pathways. The bottom panel illustrates the mechanistic rationale for preserving, and potentially facilitating, compensatory contralesional activity rather than indiscriminately suppressing the unaffected hemisphere. Such facilitatory strategies remain investigational and require further clinical validation.

### 3.1 The interhemispheric competition model

The IHC model has served as the bedrock of neuromodulation strategies for decades. Rooted in the concept of mutual inhibition, the IHC model characterizes the interaction between the two cerebral hemispheres as a dynamic rivalry mediated by the corpus callosum (30). In the healthy brain, this mutual suppression is functional and homeostatic, ensuring precise unimanual control. However, in the stroke-affected brain, this normally balanced relationship transforms into a pathological state of unilateral dominance, fundamentally altering the landscape of motor recovery.

Unimanual motor control relies on a delicate interhemispheric equilibrium rather than the isolated output of the contralateral cortex. When the primary motor cortex (M1) is activated, it transmits excitatory descending commands to the spinal cord. Simultaneously, it sends inhibitory signals to the homologous M1 in the opposite hemisphere. Anatomically, these excitatory callosal fibers terminate on local GABAergic interneurons within the target hemisphere, exerting a hyperpolarizing influence on the corticospinal output neurons (31). This mechanism, known as TCI, serves a critical function analogous to center-surround inhibition in sensory systems; it sharpens motor focus by suppressing mirror movements and preventing interference from the quiescent hemisphere (32). The strength and integrity of this inhibition are clinically quantified using TMS metrics, specifically interhemispheric inhibition (IHI) and the ipsilateral silent period (iSP). In healthy individuals, these inhibitory forces are symmetrical, maintaining a state of mutual check-and-balance that optimizes fine motor control.

The IHC model posits that a unilateral stroke catastrophically disrupts this equilibrium, subjecting the paretic limb to what is termed a double hazard (16). The primary hazard is the structural loss itself, where the ischemic or hemorrhagic lesion destroys the neural network in the ipsilesional M1 or its descending CST, directly reducing the excitatory drive to the spinal motor pool. Compounding this structural deficit is a secondary, functional hazard arising from the loss of interhemispheric counter-balance. Due to the diminished output from the damaged ipsilesional cortex, the inhibitory restraint acting upon the contralesional M1 is withdrawn. Consequently, the disinhibited contralesional hemisphere becomes hyperactive and exerts an excessive, pathological inhibitory drive back onto the already struggling ipsilesional cortex via the corpus callosum (16, 33). This recursive cycle creates a winner-takes-all scenario where the healthy hemisphere effectively acts as a neural adversary, actively suppressing the recovery of the injured side. Functional MRI studies frequently corroborate this view by demonstrating that widespread contralesional activation correlates with poor motor outcomes, interpreting such activity not as compensation but as a marker of maladaptive disinhibition (34–36).

Based on the premise that the contralesional hemisphere acts as an aggressor, the logical therapeutic intervention is to suppress it. This deduction has driven the widespread adoption of inhibitory neuromodulation protocols, most notably low-frequency rTMS applied to the contralesional M1. The physiological objective is to downregulate the excitability of the unaffected hemisphere, thereby reducing TCI and releasing the perilesional cortex from suppression. While meta-analyses have historically supported the efficacy of this approach in patients with mild-to-moderate impairment, the universality of the model has faced increasing scrutiny as clinical applications expand to more severe populations (37, 38).

Critically, the IHC model exhibits significant limitations when applied to severe stroke scenarios. It tends to oversimplify callosal function as purely inhibitory, neglecting the integrative role of callosal connections that may shift from inhibition to excitation to support function in severely damaged brains. Furthermore, a clinical paradox has emerged wherein a subset of patients, particularly those with extensive damage, exhibit functional deterioration when the hyperactive contralesional hemisphere is inhibited (39, 40). This suggests that in specific contexts of depleted structural reserve, contralesional activity may be adaptive rather than pathological. These anomalies indicate that the role of the unaffected hemisphere is not fixed but state-dependent, necessitating a theoretical pivot toward the alternative perspective known as the Vicariation model.

### 3.2 The Vicariation model

While the IHC model views the contralesional hemisphere as a competitor to be silenced, the Vicariation model views it as a surrogate to be empowered. Derived from the Latin vicarius, meaning substitute, this concept posits that spatially distinct neural areas can assume the functions of a damaged region. This perspective challenges the central axiom of the IHC framework that contralesional activity is inherently pathological. Instead, it suggests that the bilateral activation observed in stroke survivors represents an adaptive recruitment strategy akin to the bi-hemispheric mode of operation seen in early development (41). Strong evidence for this supportive role comes from virtual lesion studies. If the contralesional hemisphere were truly maladaptive, disrupting it should improve paretic limb function. However, in patients with severe motor impairment, inhibiting the contralesional motor or premotor cortex (PMC) causes significant functional deterioration (42). This deterioration provides evidence that, in at least a subset of severely impaired patients, the contralesional hemisphere may contribute functionally to paretic-limb motor output.

The viability of the Vicariation model depends on the existence of anatomical pathways capable of conveying motor commands from the unaffected hemisphere to the paretic limb. Although the crossed lateral CST typically dominates motor control, the motor system possesses latent redundancy that can be unmasked following catastrophic injury. Approximately 10 to 15% of corticospinal fibers descend ipsilaterally via the anterior CST, offering a potential route for ipsilateral control (43). Perhaps more significant for severe recovery is the corticoreticular tract (CRT). Unlike the highly lateralized corticospinal system, the CRT provides substantial bilateral innervation to spinal motor pools controlling proximal and postural muscles (44). Diffusion tensor imaging (DTI) studies confirm that in patients with chronic hemiparesis, increased fiber volume and fractional anisotropy (FA) in the contralesional CRT correlate strongly with better functional outcomes (45, 46). This suggests that in the absence of a functional ipsilesional tract, recovery is vicarious and mediated by the remodeling of these alternative, phylogenetically older pathways.

The clinical application of the Vicariation model necessitates a therapeutic strategy diametrically opposed to the IHC framework. If the contralesional hemisphere supports recovery, the logical intervention is facilitation rather than suppression. High-frequency rTMS or anodal tDCS applied to the contralesional primary motor or dorsal premotor cortex (PMd) has shown efficacy in patients who are refractory to standard inhibitory protocols. This model appears most relevant for individuals with depleted structural reserve (47). In cases where the ipsilesional CST is extensively disrupted, the brain relies on the contralesional hemisphere to drive function. Consequently, strictly adhering to the IHC dogma by inhibiting the contralesional cortex in such patients may paradoxically worsen outcomes by eliminating the primary compensatory mechanism remaining available to the motor system.

## 4 Dialectical reconciliation: from conflict to synthesis

The persistent dichotomy between the IHC model and the Vicariation model suggests that neither framework possesses the comprehensive explanatory power required to account for the full spectrum of stroke recovery. The clinical heterogeneity observed in rTMS trials is not merely a statistical artifact; rather, it reflects distinct pathophysiological states that exist along a continuum of severity. To resolve this, we must move beyond viewing these models as competing binary options. Instead, they should be understood as complementary mechanisms that become dominant at different thresholds of structural integrity. This chapter delineates the theoretical bridge between these opposing views. It critiques initial attempts at unification and proposes a refined dialectical synthesis based on the interaction between residual anatomy and functional network reorganization.

### 4.1 Critique of the bimodal balance-recovery model

The recognition that a single model cannot explain recovery across all patient stratifications led to the formulation of the Bimodal Balance-Recovery Model. Proposed by Di Pino and colleagues in a seminal framework, this model introduced the concept of “structural reserve” as a governing variable that dictates whether bi-hemispheric interactions facilitate or impede functional restoration (20). The model posits that IHI is only maladaptive in patients with high structural reserve who retain significant CST integrity. Conversely, in patients with low structural reserve and extensive damage, the model suggests that contralesional activity shifts from a competitive to a supportive role. While this hypothesis provided a necessary theoretical compromise, its translation into clinical utility remains constrained by several critical limitations.

The primary limitation of the Bimodal Balance-Recovery Model is its qualitative nature. Although it identifies structural reserve as the independent variable, it functions as a heuristic device rather than a quantitative algorithm (48). The model describes a theoretical tipping point where the role of the unaffected hemisphere inverts, yet it fails to establish the precise neurophysiological cut-off points required to locate this transition in an individual patient. Clinicians are left with a conceptual understanding that severity influences mechanisms but lack specific diagnostic criteria to determine if a patient falls within the IHC-dominant or Vicariation-dominant spectrum. This ambiguity perpetuates the trial-and-error approach currently seen in rTMS prescription.

Furthermore, the Bimodal Balance framework focuses disproportionately on the CST while underestimating the complexity of alternative motor networks. It largely frames the vicariation component as the unmasking of ipsilateral corticospinal projections or homologous area recruitment (49). This perspective neglects the crucial role of the reticulospinal tract (RST) and the cortico-reticulospinal circuit, which are phylogenetically older pathways essential for synergistic movement and spasticity modulation in severe stroke (50). The model assumes that the contralesional hemisphere compensates primarily through homologous motor areas, failing to fully account for the recruitment of secondary motor areas such as the PMd or the supplementary motor area (SMA) (51). These regions exhibit distinct interhemispheric connectivity patterns that do not align strictly with the binary inhibition/facilitation dichotomy proposed by Di Pino.

Finally, the Bimodal Balance framework treats the transition between inhibition and vicariation as a static trait determined solely by lesion load. It often fails to integrate the temporal dynamics of post-stroke reorganization (52). The role of the contralesional hemisphere is not fixed by the initial injury severity alone; it evolves longitudinally as the brain transitions from the acute phase of diaschisis to the chronic phase of network consolidation (53). A patient might exhibit a vicariation-dependent state in the early weeks post-stroke but transition toward an IHC-dominant state as perilesional networks reorganize. The static nature of the current model lacks the temporal resolution to guide phase-specific rTMS interventions. More recent network-based perspectives further suggest that structural reserve should not be regarded as a single global property of the motor system. Rather, reserve may be region-specific, reflecting differential preservation of the ipsilesional M1 and its corticospinal output, secondary motor regions such as the PMd and SMA, and alternative descending pathways such as the CRT. Consequently, similar degrees of CST injury may coexist with different residual network architectures and compensatory capacities. Therefore, a more rigorous dialectical synthesis is required to link specific physiological biomarkers to the mechanistic switch between these two recovery modes. Importantly, however, the Bimodal Balance-Recovery Model was conceived primarily as a mechanistic framework rather than an operational treatment-selection algorithm; it does not specify a stepwise biomarker pathway for translating structural reserve into individualized neuromodulation strategies.

### 4.2 Dialectical synthesis of mechanisms

To achieve a true synthesis of the IHC and Vicariation models, we must deconstruct the paradox to reveal that the perceived conflict is actually a coherent biological response to varying degrees of network failure. The brain does not choose between competition and vicariation arbitrarily; it defaults to the most efficient available pathway based on the preservation of the corticospinal scaffold (7). We propose a dialectical synthesis in which the status of the ipsilesional CST serves as a principal physiological axis, while the functional contribution of the contralesional hemisphere is further shaped by the residual organization of secondary motor regions and alternative descending pathways.

In patients with mild-to-moderate hemiparesis, the ipsilesional CST retains sufficient fiber density to conduct descending volleys to the spinal motor pool (54). In this physiological context, the motor system attempts to restore the canonical, crossed control pattern. The healthy brain relies on TCI to sharpen motor commands and suppress mirror activity, a mechanism necessary for independent digit movement. Following a minor stroke, the surviving neural resources in the ipsilesional M1 are engaged in a struggle to regain control but are actively suppressed by the disinhibited contralesional M1 (16). Here, the IHC model is accurate because the contralesional activity is effectively noise that drowns out the weakened signal from the lesioned hemisphere (55). The inhibition from the healthy side adds a functional burden to the structural deficit. Consequently, applying low-frequency rTMS to the unaffected hemisphere is therapeutic because it restores the signal-to-noise ratio and allows the preserved ipsilesional tract to resume its role as the primary driver of movement (56).

The physiological landscape changes fundamentally when the ipsilesional CST is severed or critically attenuated, a state corresponding to the Vicariation model. In severe stroke, the canonical crossed pathway is no longer viable as a conduit for motor execution (57). The brain detects this failure and initiates a fallback protocol by recruiting the contralesional hemisphere and alternative descending pathways such as the cortico-RST (58). Under these conditions, the high excitability observed in the contralesional M1 is not a pathological source of inhibition; rather, it is a compensatory drive required to energize these alternative pathways (59). The contralesional hemisphere may assume an increasingly important compensatory role in residual motor output. In this context, indiscriminate inhibitory stimulation may be detrimental if it suppresses networks contributing to compensatory motor control (60). This may help explain the clinical observation that inhibitory protocols can produce functional deterioration in some severely impaired patients (61).

The reconciliation of these models lies in understanding that they describe the behavior of the motor system at opposite ends of a structural continuum. The transition from the IHC state to the Vicariation state is likely non-linear and defined by a critical threshold of CST integrity (59). However, this transition is unlikely to be determined by CST integrity alone; the effective threshold may vary according to lesion location, the preservation of secondary motor regions, and the availability of alternative descending pathways. Above the effective reserve threshold, the ipsilesional cortex can still drive the spinal cord; therefore, IHC is the dominant impediment. Below this effective reserve threshold, the ipsilesional cortex loses sufficient connectivity to the spinal cord; therefore, the system increasingly relies on vicarious compensation (62).

This synthesis resolves the theoretical conflict by reframing the dichotomy of the unaffected hemisphere as a state-dependent variable. The unaffected hemisphere acts as an aggressor only when it competes with a viable ipsilesional tract, whereas it functions as a savior when the ipsilesional tract is functionally incompetent. This realization exposes the fundamental flaw in the one-size-fits-all approach to rTMS. The efficacy of neuromodulation may depend, in part, on accurately characterizing the patient’s position along this continuum (47). Treating a low-reserve patient with an IHC-based protocol effectively dismantles their scaffold for recovery, while treating a high-reserve patient with a Vicariation-based protocol may reinforce maladaptive mirror movements and limit fine motor recovery.

Consequently, the critical clinical imperative is to identify the biomarkers that define this threshold. We must move beyond clinical scales, which are surrogate markers of function, and utilize physiological measures of structural integrity. This necessitates the integration of MEP and DTI into the decision-making process. These metrics provide complementary anatomical and physiological information for estimating structural reserve. By doing so, we can categorize patients not merely by symptoms but by the underlying mechanism of their residual motor control. This forms the logical basis for the stratified decision framework detailed in the subsequent section (see Figure 2).

**Figure 2:**
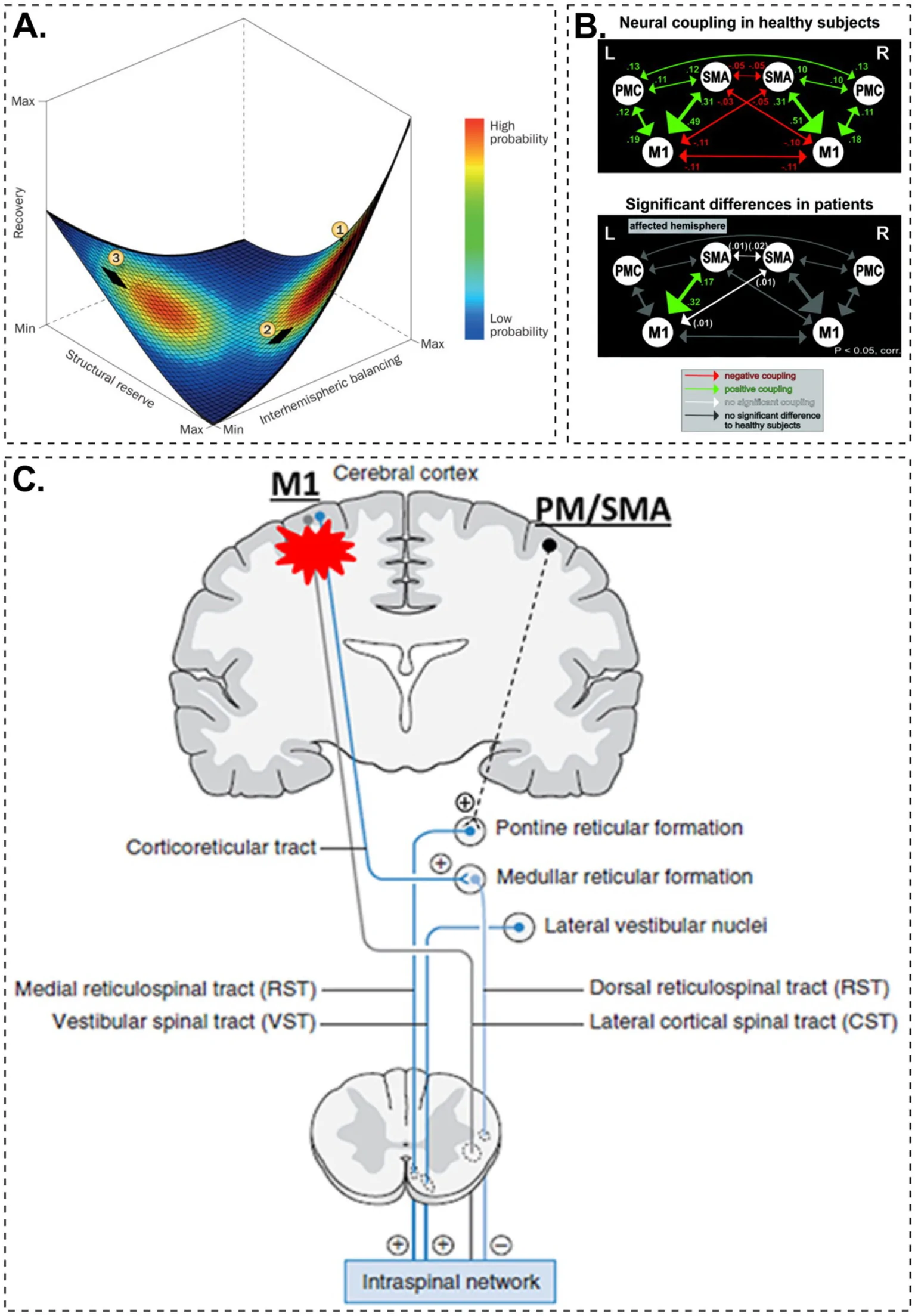
Integrating structural reserve, network connectivity, and anatomical compensation. **(A)** The Bimodal Balance-Recovery Model (20). **(B)** Network complexity beyond interhemispheric competition (52). Dynamic causal modeling (DCM) reveals that stroke-induced reorganization extends beyond the M1-M1 axis. Stroke patients exhibit pathological inhibitory coupling (red arrow) from the contralesional M1, but also demonstrate altered effective connectivity involving secondary motor areas such as the supplementary motor area (SMA) and premotor cortex (PMC). This highlights the network complexity often oversimplified by static bimodal models. **(C)** The anatomical substrate of vicariation. A schematic comparison of descending motor pathways (50).

## 5 Decision-making fulcrum: structural reserve assessment

The transition from a generalized, one-size-fits-all approach in neurorehabilitation to a precision medicine paradigm necessitates the identification of a physiological pivot point, which is a fulcrum upon which therapeutic decisions can be balanced. The dialectical synthesis of the IHC model and the Vicariation model suggests that these are not mutually exclusive theories but rather distinct operational modes of the post-stroke brain, dictated by the residual architecture of the motor network. This residual capacity is conceptually defined as structural reserve. Within the present framework, structural reserve is considered a multidimensional property centered on CST integrity but modulated by the preservation and reorganization of distributed motor regions and alternative descending pathways. We posit that structural reserve serves as a central organizing construct for patient stratification, informing how the functional contribution of the contralesional hemisphere may be interpreted within the broader residual motor network. This chapter provides an exhaustive analysis of the structural reserve assessment framework, dissecting the microstructural hierarchy of the CST, the neurophysiological characterization of MEP, and the algorithmic integration of these multimodal data streams to inform individualized rTMS strategy selection.

### 5.1 The hierarchy of CST integrity

The CST constitutes the principal anatomical substrate for voluntary motor execution in humans, mediating fine digital dexterity and independent limb control. Its structural integrity following a cerebrovascular accident is universally acknowledged as the single most robust predictor of upper extremity motor recovery potential (54). However, treating CST integrity as a binary variable, that is, intact versus severed, is fundamentally insufficient for the nuanced stratification required in precision rTMS. Recent advances in DTI and high-angular resolution diffusion imaging (HARDI) have necessitated a hierarchical classification that accounts for FA thresholds, the topography of Wallerian degeneration, and the crucial, often overlooked role of non-pyramidal pathways like the CRT (46). To accurately situate a patient on the recovery spectrum between IHC-dominance and Vicariation-dominance, clinicians must evaluate the CST within a graded hierarchy of microstructural preservation.

DTI provides a non-invasive window into the microstructural architecture of white matter by quantifying the diffusion properties of water molecules. In healthy, highly organized white matter tracts like the CST, water diffusion is restricted perpendicular to the axonal fibers by myelin sheaths and axonal membranes, resulting in high anisotropy. Following ischemic or hemorrhagic injury, the breakdown of axonal membranes, demyelination, and cytoskeletal disintegration lead to more isotropic diffusion. This phenomenon manifests quantitatively as decreased FA and increased mean diffusivity (MD) or radial diffusivity (RD) (63). Research over the past decade has established specific DTI metrics, particularly the FA asymmetry index (FA_asym) or ratio (rFA), typically calculated at the posterior limb of the internal capsule (PLIC), as potent surrogates for structural reserve (64). The PLIC serves as an ideal region of interest for this assessment because it represents an anatomical bottleneck where descending motor fibers are densely packed, rendering them highly susceptible to quantification of injury burden relative to more dispersed cortical regions (65).

The hierarchy of CST integrity implies a stratification of injury severity that correlates with distinct mechanisms of recovery. This hierarchy can be conceptualized through a multi-tiered model, heavily informed by the seminal work of Lindenberg (66). This 3-Tier classification system distinguishes between damage to the direct pyramidal tract versus alternate motor fibers, providing a granular framework that is essential for resolving the IHC-Vicariation paradox.

Patients in Tier 1, characterized by high structural reserve with an intact or minimally damaged CST, exhibit high FA values in the ipsilesional PLIC, typically maintaining an FA asymmetry index of less than 0.15 or an rFA greater than 0.8 (62). In these individuals, the structural continuity between the M1 and the spinal cord remains largely preserved, with observed hemiparesis frequently attributable to functional diaschisis, perilesional edema, or reversible metabolic suppression rather than irreversible axonal transection; neurophysiologically, this state supports the Canonical Recovery mechanism where the ipsilesional hemisphere retains the capacity to drive motor output (7). Empirical studies indicate that in this stratum, the contralesional hemisphere frequently exerts maladaptive IHI via the corpus callosum, making the structurally intact but functionally suppressed ipsilesional M1 the “victim” of this inhibition. Consequently, patients in Tier 1 are the prime candidates for classic IHC-based inhibitory rTMS protocols applied to the contralesional M1, as suppressing the healthy hemisphere releases the preserved ipsilesional network from pathological inhibition and allows it to regain control over spinal motoneurons without compromising the overall motor drive (17).

Tier 2 represents the “gray zone” of stroke recovery where rTMS decision-making becomes most complex, with patients typically presenting with FA asymmetry indices between 0.15 and 0.25 or an rFA between 0.5 and 0.8 (48, 62). Structurally, a significant proportion of CST fibers have undergone Wallerian degeneration, yet a residual population remains viable. In this context, the interplay between IHC and vicariation is dynamic and often state-dependent in that inhibiting the contralesional hemisphere might be beneficial for patients at the upper end of this spectrum, while those closer to the lower bound of integrity may see the contralesional hemisphere already transitioning towards a supportive role. Thus, the decision to inhibit or facilitate requires integrating DTI data with functional biomarkers as structural imaging alone may not reveal whether the residual fibers are physiologically functional or dormant. The breakdown of microstructure in this Tier suggests that while the direct corticomotoneuronal pathway is compromised it is not obliterated, necessitating a therapeutic strategy that carefully balances disinhibition of the ipsilesional side with the preservation of any compensatory drive from the contralesional side.

Finally, patients with Tier 3 exhibit severe disruption of the CST, characterized by an FA asymmetry index exceeding 0.25 or an rFA dropping below 0.5 (62). In many cases, probabilistic tractography fails to reconstruct the ipsilesional CST entirely due to the loss of coherent diffusion vectors. Under these conditions, the canonical crossed corticospinal pathway is functionally incompetent to support independent muscle activation. The central nervous system, detecting this catastrophic failure, defaults to a vicarious recovery mode by recruiting alternative descending pathways. The most significant of these is the CRT. Originating primarily from the PMC and SMA, the CRT descends to the reticular formation in the brainstem and influences spinal motor neurons bilaterally via the RST. Unlike the highly lateralized CST, the CRT provides substantial bi-hemispheric innervation, particularly to proximal and postural muscles (67).

DTI tractography studies have demonstrated that in patients with complete CST disruption, the preserved integrity of the contralesional CRT correlates positively with residual motor function and gait recovery (44). This finding provides the anatomical substrate for the Vicariation model: when the ipsilesional CST is destroyed, the brain recruits the contralesional hemisphere to drive the paretic limb via the uncrossed, polysynaptic CRT/RST pathway. In this Tier, strictly adhering to the IHC dogma by inhibiting the contralesional premotor areas would theoretically sever the only remaining line of communication to the paretic limb, potentially precipitating a deterioration in functional status^47^. Instead, these findings provide a physiological rationale for considering strategies that preserve or facilitate compensatory contralesional networks rather than routinely suppressing them. Identification of a low-reserve profile should therefore prompt particular caution regarding standard contralesional inhibitory protocols.

Despite their value for characterizing CST injury, DTI-derived FA measures should not be interpreted as direct or universally standardized measures of structural reserve. FA is a composite microstructural metric that can be influenced by factors beyond axonal integrity, including edema, crossing-fiber architecture, age-related white matter changes, lesion location, and partial-volume effects. In addition, absolute FA values and derived asymmetry or ratio measures may vary according to scanner field strength, diffusion acquisition parameters, preprocessing pipelines, region-of-interest definition, and tractography methodology. Consequently, thresholds reported in individual cohorts should not be assumed to represent universally transferable biological cutoffs across centers or stages of stroke recovery. Within SPiRe, FA-based measures are therefore most appropriately interpreted as complementary indicators of CST preservation in conjunction with clinical and neurophysiological evidence.

Beyond these methodological sources of variability, the temporal evolution of DTI metrics is also critical for interpretation and stratification. While acute DTI lesions represent direct ischemic injury and cytotoxic edema, chronic FA reductions reflect secondary Wallerian degeneration extending retrograde into the cortex and anterograde into the cerebral peduncle and pons (68). Studies have shown that rFA values measured at 30 days are independent predictors of long-term motor outcome at two years, suggesting that the window for determining structural reserve remains open into the subacute phase (69). Furthermore, the specific pattern of tract involvement, in terms of whether the lesion affects the PLIC, the corona radiata, or the brainstem, differentially impacts the potential for recovery and the dominance of the CRT. Therefore, assessing the hierarchy of tract integrity involves determining whether the patient relies on the remnant CST (Tier 1/2) or has shifted to the vicarious CRT (Tier 3), which constitutes the first fundamental step in the decision-making fulcrum. Accordingly, longitudinal change and within-patient asymmetry may be more informative than isolated absolute FA values when interpreting structural reserve across different stages of recovery.

### 5.2 Motor evoked potential characteristics

While DTI delineates the anatomical roadmap of structural reserve, MEP elicited by TMS provide a functional readout of the tract’s physiological conductivity. The integration of MEP characteristics into the stratification framework is indispensable because anatomical preservation does not always equate to functional viability. rTMS exerts its therapeutic effects by modulating neuronal excitability; thus, determining the baseline excitability status of the motor pathway is critical for predicting the response to stimulation. The physiological characterization of MEPs focuses on three primary dimensions: presence versus absence, latency characteristics, and amplitude dynamics, each serving as a vital node in the decision-making process for rTMS targeting (27).

MEP status serves as an important functional stratification marker in this context, as the presence or absence of an MEP in the paretic limb following stimulation of the ipsilesional M1 provides clinically relevant information regarding residual corticospinal excitability. This status acts as a key component in prognostic algorithms such as the PREP2 model, offering a high negative predictive value for functional recovery (62, 64). For MEP-positive patients, the elicitation of a reproducible MEP implies that a functional pathway from the cortex to the muscle remains viable. This finding correlates strongly with the IHC model’s applicability, as it suggests the ipsilesional CST is capable of transmitting descending volleys if released from IHI. Such patients typically exhibit proportional recovery patterns and respond well to inhibitory rTMS of the contralesional hemisphere or excitatory rTMS of the ipsilesional hemisphere, with sufficient structural reserve to support a restitution-based strategy (48). In contrast, MEP-negative status, particularly when assessed with high-output stimulation and facilitation maneuvers, signifies a critical depletion of physiological structural reserve. In these cases, the signal from the ipsilesional M1 is effectively blocked, or the motor threshold is pathologically elevated beyond the stimulator’s capacity, and this status is a strong indicator that the patient may be reliant on vicarious pathways for any residual movement. The application of standard inhibitory rTMS to the unaffected hemisphere in MEP-negative patients carries the significant risk of suppressing the compensatory drive, potentially leading to worse functional outcomes or impeding the recruitment of alternative networks (47, 59).

However, MEP absence should not be interpreted as synonymous with complete anatomical disruption of the CST. The ability to elicit an MEP depends not only on tract integrity but also on methodological and physiological factors, including stimulation intensity, coil position and orientation, target muscle selection, background muscle activation, cortical excitability, and the timing of assessment after stroke. In severely impaired patients, an absent response may therefore reflect markedly elevated motor thresholds or transient physiological suppression rather than irreversible structural disconnection. Conversely, MEP presence confirms functional corticospinal output at the time of testing but does not, by itself, establish which broader recovery mechanism is dominant. Accordingly, MEP status should be interpreted in conjunction with the clinical phenotype, structural imaging, and other neurophysiological findings rather than as an isolated binary determinant of SPiRe classification.

Beyond the simple presence or absence of a response, the onset latency of the MEP provides a sophisticated means to discriminate which pathway is mediating the recovery. An MEP with normal or slightly delayed latency, comparable to the unaffected side or within a < 2–3 ms difference, indicates conduction via the fast, monosynaptic corticomotoneuronal connections of the preserved CST. This physiological signature is compatible with a Tier 1 or upper Tier 2 profile, validating the use of strategies that reinforce the ipsilesional M1. In severe stroke, MEPs, when present, are typically characterized by significantly prolonged latencies. The Vicariation model posits that these alternative pathways often originate from the contralesional hemisphere or secondary motor areas, so a significantly prolonged latency may provide supportive evidence of greater reliance on alternative descending pathways. In such cases, the findings may provide a physiological rationale for considering strategies that preserve or facilitate alternative descending pathways. Conversely, indiscriminate inhibition of the contralesional hemisphere may warrant caution when convergent evidence suggests that contralesional networks contribute to residual motor output (47).

Another critical dimension of MEP assessment is amplitude dynamics and the presence of ipsilateral responses, as these metrics reflect the “gain” or total neural drive of the motor system. MEP amplitude, typically expressed as a ratio to the unaffected side or to the compound muscle action potential, correlates with the loss of corticospinal neurons, while the detection of ipsilateral MEPs (iMEPs), which are responses elicited in the paretic limb by stimulating the contralesional hemisphere, may provide evidence of increased ipsilateral or alternative descending pathway recruitment (70). In healthy adults, iMEPs are rare or require high-intensity stimulation to elicit, whereas their emergence after stroke may reflect the unmasking of uncrossed corticospinal fibers or increased recruitment of bilaterally projecting reticulospinal pathways (58). In patients with severe motor impairment, the presence of iMEPs may support the interpretation that the contralesional hemisphere contributes to residual paretic-limb motor output. When considered together with severe CST disruption and the broader clinical phenotype, this finding raises particular concern regarding indiscriminate inhibitory stimulation of the contralesional hemisphere and may provide a physiological rationale for investigating facilitatory strategies targeting compensatory networks. Conversely, in patients with relatively preserved CST integrity, ipsilateral responses may have a different functional significance and should therefore not be interpreted independently of the broader residual motor architecture. The same interpretive caution applies to MEP latency and iMEP detection, both of which are influenced by stimulation parameters and may reflect overlapping corticospinal, ipsilateral corticospinal, and cortico-reticulospinal contributions rather than a single anatomically specific pathway. Their principal value within SPiRe is therefore complementary: they refine the interpretation of residual motor-system organization when considered together with MEP status and structural evidence.

By systematically evaluating these three dimensions, namely status, latency, and ipsilateral connectivity, MEP assessment can provide complementary functional information for estimating residual motor-system organization and informing subsequent rTMS strategy selection.

### 5.3 Multimodal assessment integration

The culmination of structural reserve assessment is the integration of CST hierarchy and MEP characteristics into a cohesive, multimodal decision-making framework. Historically, prediction tools such as the PREP2 algorithm have successfully combined clinical scores (Shoulder Abduction and Finger Extension, SAFE), MEP status, and neuroimaging measures to prognosticate upper-limb motor outcomes after stroke (71–73). More recent stratification approaches, including the Stratification Algorithm for Repetitive TMS in Stroke (START), have further advanced the field by incorporating structural and neurophysiological characteristics into the selection and evaluation of rTMS interventions (72–74). The proposed SPiRe framework builds on, rather than replaces, these preceding models. Its incremental contribution lies in explicitly linking structural reserve to the competing IHC and Vicariation mechanisms and extending stratification beyond the conventional interaction between the ipsilesional and contralesional M1 to incorporate compensatory secondary motor regions and alternative descending pathways. Accordingly, whereas the Bimodal Balance-Recovery Model provides the mechanistic concept of structural reserve, PREP2 primarily addresses prognosis, and START advances biomarker-informed rTMS stratification, SPiRe integrates these concepts into a mechanism-oriented framework designed to relate the inferred residual motor architecture to candidate neuromodulation targets and directions. Therefore, we propose the Stroke Precision in Reservoir-guided Neuromodulation (SPiRe) framework to translate this mechanistic integration into biomarker-informed neuromodulation target selection (75).

The operational application of SPiRe begins with consideration of the timing of assessment. For patients evaluated in the acute phase, the SAFE score provides a practical initial clinical screen, as a SAFE score ≥ 5 (out of 10) within 72 h post-stroke is associated with MEP-positive status and relatively preserved CST integrity (76, 77). However, this early SAFE threshold should not be regarded as a time-invariant surrogate of structural reserve. For patients first evaluated during the subacute or chronic phase, stratification should instead begin with the contemporaneous motor phenotype, because motor function and network organization may evolve substantially after the acute phase as edema and diaschisis resolve, Wallerian degeneration progresses, and compensatory networks reorganize. Accordingly, a SAFE score < 5 obtained months after stroke should not automatically be assigned the same structural meaning as the same score obtained within the first 72 h (78, 79).

Following clinical assessment, standard TMS-based MEP testing provides the next functional stratification step. In acutely assessed patients with SAFE < 5, and in later-stage patients whose current motor phenotype suggests substantial impairment, the presence of an ipsilesional MEP provides evidence that functional corticospinal output remains detectable. Conversely, MEP absence increases concern for severely impaired corticospinal function but, as discussed above, should not be equated with complete anatomical CST disruption (80). DTI therefore serves as an additional structural arbitration step when MEPs are absent or when clinical and neurophysiological findings are discordant (71). This sequence prioritizes readily obtainable clinical and physiological information before escalating to advanced structural imaging.

Within this hierarchy, confidence in mechanistic assignment depends on concordance across clinical, neurophysiological, and structural evidence. A relatively preserved clinical phenotype together with detectable ipsilesional MEPs and preserved CST structure supports a higher-reserve profile consistent with Tier 1 or the upper range of Tier 2. At the opposite end of the spectrum, severe motor impairment, absent ipsilesional MEPs, and marked CST disruption on DTI provide convergent evidence for a low-reserve profile consistent with Tier 3. Advanced TMS findings, including prolonged-latency responses or iMEPs, may provide additional evidence regarding the recruitment of alternative descending pathways when available. Thus, individual biomarkers are not intended to assign a Tier in isolation; rather, confidence increases when independent clinical, physiological, and anatomical measures converge on the same residual motor architecture.

Discordant findings should reduce, rather than artificially increase, the certainty of Tier assignment. For example, an absent ipsilesional MEP in the presence of partial CST preservation on DTI should be regarded as a discordant or indeterminate profile rather than automatically classified as Tier 3. Such discordance may reflect physiologically inactive but structurally preserved fibers, transient cortical suppression, methodological limitations of MEP testing, or uncertainty inherent in DTI-derived measures. These patients therefore constitute an important component of the SPiRe “gray zone,” in which definitive mechanistic assignment should be deferred pending additional or longitudinal evidence.

For patients within this gray zone, repeated assessment over time may help determine whether clinical, neurophysiological, and structural measures converge as recovery evolves. Exploratory probe sessions may also be considered as a hypothesis-generating approach, with short-term changes in MEP amplitude or cortical excitability, standardized motor performance, and task-based or kinematic measures serving as potential response readouts. However, no validated single-session threshold currently exists for defining a clinically meaningful response or for predicting the optimal long-term rTMS protocol. Probe-session findings should therefore be interpreted as exploratory physiological information rather than as a definitive treatment-selection test. Future refinement of this process may also incorporate multivariable or machine-learning approaches that integrate lesion characteristics, FA measures, and neurophysiological features to estimate individual responsiveness (81).

Importantly, the full SPiRe pathway assumes access to multimodal assessment, but such resources are not universally available. In settings without DTI or advanced TMS measures such as iMEPs, a simplified fallback pathway can rely on the current clinical motor phenotype and standard ipsilesional MEP testing as the minimum practical assessment. Patients with relatively preserved function and reproducible ipsilesional MEPs may be provisionally considered to have greater functional reserve, whereas patients with severe impairment and absent MEPs should be regarded as having uncertain or potentially low reserve rather than being automatically assigned to Tier 3. When advanced biomarkers are unavailable, the framework should preserve this uncertainty and favor longitudinal reassessment or referral for additional evaluation when clinically feasible. Thus, limited resource availability reduces the confidence of mechanistic assignment but does not preclude use of the broader SPiRe reasoning framework.

Taken together, SPiRe uses structural reserve as an organizing principle for integrating clinical, neurophysiological, and anatomical evidence rather than treating any single biomarker as determinative. The framework is designed to estimate the most plausible residual motor architecture and the likely functional contribution of the contralesional hemisphere, while explicitly preserving uncertainty when biomarkers are discordant, unavailable, or obtained at different stages of recovery. Greater biomarker concordance increases confidence in mechanistic stratification, whereas discordance or missing information should prompt more cautious interpretation and, where appropriate, reassessment. This graded-confidence approach provides the basis for translating multimodal assessment into individualized neuromodulation strategy selection (see Table 2).

**Table 2:** Resource-adaptive multimodal assessment pathway for SPiRe-guided rTMS stratification in post-stroke motor recovery.

Step	Assessment	Finding	Interpretation within SPiRe	Next step/implication
1. Clinical entry	Early SAFE score (acute phase)	≥5 within 72 h	Greater likelihood of preserved functional reserve	Supports initial higher-reserve classification; functional confirmation may be obtained when clinically indicated
1. Clinical entry	Early SAFE score (acute phase)	<5 within 72 h	Reduced or uncertain functional reserve; clinical severity alone does not establish CST integrity	Proceed to standard ipsilesional MEP assessment
1. Clinical entry	Current motor phenotype (subacute or chronic phase)	Current degree and pattern of motor impairment	Early SAFE thresholds should not be directly extrapolated to later-stage assessment because motor function and network organization evolve over time	Base stratification on contemporaneous clinical findings and proceed to current neurophysiological assessment
2. Functional assessment	Standard ipsilesional MEP	Present (MEP+)	Detectable functional corticospinal output; supports preserved or intermediate functional reserve	Integrate with the clinical phenotype; structural confirmation may be considered when findings remain uncertain or discordant
2. Functional assessment	Standard ipsilesional MEP	Absent (MEP−)	Severe corticospinal dysfunction is possible, but MEP absence does not establish complete anatomical CST disruption	Proceed to structural arbitration with DTI when available
3. Structural arbitration	DTI assessment of CST integrity, including PLIC FA measures	Relatively preserved CST integrity	Structural evidence supports preserved reserve	Integrate with clinical and MEP findings to assess biomarker concordance
3. Structural arbitration	DTI assessment of CST integrity, including PLIC FA measures	Partial CST preservation	Intermediate structural reserve; physiological significance remains uncertain if MEP is absent	Evaluate concordance with MEP and other available physiological evidence; consider a gray-zone classification if discordant
3. Structural arbitration	DTI assessment of CST integrity, including PLIC FA measures	Severe CST disruption	Structural evidence supports low reserve	Assess clinical and neurophysiological evidence for compensatory network recruitment
4. Advanced refinement	Advanced TMS and alternative-pathway assessment	Normal or mildly delayed MEP latency with detectable ipsilesional output	Supports relatively efficient conduction through residual corticospinal pathways	Strengthens evidence for a restitution-oriented residual motor architecture
4. Advanced refinement	Advanced TMS and alternative-pathway assessment	Prolonged MEP latency and/or detectable iMEP	May indicate recruitment of slower, ipsilateral, or polysynaptic descending pathways	Interpret together with CST integrity and clinical phenotype to refine the inferred recovery mechanism
4. Advanced refinement	Alternative descending pathway assessment	Evidence of preserved or reinforced CRT/RST organization	Supports the availability of alternative descending pathways and possible compensatory recruitment	Incorporate into network-level interpretation, particularly when ipsilesional CST reserve is severely reduced
5. Concordant profile	Integrated clinical, MEP, and structural evidence	Preserved clinical function, detectable ipsilesional MEP, and relatively preserved CST	Higher-confidence higher-reserve profile, broadly consistent with Tier 1 or upper Tier 2	Use the convergent biomarker profile to inform subsequent neuromodulation strategy selection
5. Concordant profile	Integrated clinical, MEP, and structural evidence	Severe motor impairment, absent ipsilesional MEP, and marked CST disruption	Higher-confidence low-reserve profile, broadly consistent with Tier 3	Evaluate evidence of compensatory network recruitment before subsequent neuromodulation strategy selection
6. Discordant profile / SPiRe gray zone	Integrated multimodal assessment	Example: MEP − despite partial CST preservation on DTI	Indeterminate profile; anatomical preservation and physiological output are discordant	Do not force Tier 2 or Tier 3 assignment; proceed to longitudinal reassessment and/or exploratory physiological testing
6. Discordant profile / SPiRe gray zone	Longitudinal reassessment	Evolution or convergence of clinical, MEP, and/or structural findings	Increasing concordance may improve confidence in the inferred residual motor architecture	Reassess mechanistic classification as recovery evolves
6. Discordant profile / SPiRe gray zone	Exploratory rTMS probe session	Short-term changes in MEP amplitude or cortical excitability, standardized motor performance, and/or task-based or kinematic measures	Exploratory evidence of physiological responsiveness; no validated single-session response threshold currently exists	Use as hypothesis-generating information rather than a definitive treatment-selection test
7. Resource-limited fallback pathway	Current clinical motor phenotype + standard ipsilesional MEP	Relatively preserved function with reproducible MEP	Provisional evidence of greater functional reserve when advanced biomarkers are unavailable	Continue clinical and neurophysiological follow-up; advanced assessment may be obtained if clinically indicated
7. Resource-limited fallback pathway	Current clinical motor phenotype + standard ipsilesional MEP	Severe impairment with absent MEP	Uncertain or potentially low reserve; insufficient evidence for confident Tier 3 assignment without structural or advanced physiological information	Preserve uncertainty; perform longitudinal reassessment or refer for advanced evaluation when clinically feasible

CRT, corticoreticular tract; CST, corticospinal tract; DTI, diffusion tensor imaging; FA, fractional anisotropy; iMEP, ipsilateral motor evoked potential; MEP, motor evoked potential; MEP−, motor evoked potential absent; MEP+, motor evoked potential present; PLIC, posterior limb of the internal capsule; RST, reticulospinal tract; rTMS, repetitive transcranial magnetic stimulation; SAFE, Shoulder Abduction and Finger Extension; SPiRe, Stroke Precision in Reservoir-guided Neuromodulation; TMS, transcranial magnetic stimulation. SAFE thresholds refer primarily to early post-stroke assessment and should not be directly extrapolated to patients first assessed during the subacute or chronic phase. DTI-derived FA thresholds should be regarded as literature-derived reference ranges rather than universally validated biological cutoffs, because their interpretation may vary with the timing of assessment, acquisition and processing methods, and patient-specific factors. MEP and DTI findings should be interpreted as complementary evidence rather than isolated determinants of Tier assignment. Greater concordance among clinical, neurophysiological, and structural findings increases confidence in the inferred residual motor architecture, whereas discordant or unavailable biomarkers should reduce confidence and preserve an indeterminate or gray-zone classification rather than force deterministic assignment. Advanced TMS measures and assessments of alternative descending pathways are intended to refine, rather than independently determine, mechanistic classification. No validated single-session response threshold currently exists for exploratory rTMS probe sessions; therefore, probe-session findings should be considered hypothesis-generating rather than definitive treatment-selection criteria. In resource-limited settings where DTI or advanced TMS measures are unavailable, the current clinical motor phenotype combined with standard ipsilesional MEP assessment constitutes the minimum practical fallback pathway, with longitudinal reassessment or referral for advanced evaluation when clinically feasible.

## 6 Emerging modalities: synergies and future directions

The preceding analysis of the IHC and Vicariation models has established a critical theoretical synthesis: the efficacy of neuromodulation is not intrinsic to the stimulation protocol itself but may depend substantially on the patient’s residual motor-system architecture and structural reserve. Having defined the “decision-making fulcrum” based on CST integrity and MEP characteristics, we must now examine how next-generation therapeutic modalities can be integrated into this stratified framework. Conventional rTMS, while foundational, often relies on tonic stimulation paradigms that may lack the temporal precision required to induce robust synaptic plasticity in resistant neural networks. The evolution of non-invasive brain stimulation is currently witnessing a transition toward sophisticated temporal patterning, multi-nodal network targeting, and closed-loop systems that synchronize central stimulation with peripheral feedback. This chapter provides an exhaustive, dialectical examination of these emerging modalities, namely TBS, multi-target stimulation, and peripheral-central combined stimulation. We analyze their neurophysiological mechanisms through the lens of the structural reserve hierarchy to examine how they might be integrated within biomarker-informed neuromodulation strategies.

### 6.1 Theta-burst stimulation applications

TBS represents a fundamental paradigm shift in the application of electromagnetic induction for neurorehabilitation. Unlike standard rTMS, which delivers evenly spaced pulses at fixed frequencies, TBS utilizes a complex temporal structure modeled after the intrinsic firing patterns of hippocampal neurons during memory consolidation and LTP (82). By delivering triplets of 50 Hz pulses repeated at a theta frequency of 5 Hz, TBS mimics the natural theta-gamma coupling of the brain, a mechanism central to synaptic efficiency and information storage. This biomimetic patterning allows TBS to induce profound changes in cortical excitability with significantly lower stimulation intensities and shorter durations (typically 190 s) compared to the 20–30 min required for conventional rTMS (83). However, the increased potency of TBS introduces a higher degree of complexity in its interaction with the stroke-injured brain, necessitating a rigorous stratification strategy to avoid maladaptive plasticity.

The operational dichotomy of TBS is traditionally divided into intermittent theta-burst stimulation (iTBS), which facilitates cortical excitability via LTP-like mechanisms, and continuous theta-burst stimulation (cTBS), which suppresses excitability via LTD-like mechanisms (84). The induction of these plastic changes is mediated by N-methyl-D-aspartate (NMDA) receptor kinetics and the modulation of calcium influx into the postsynaptic neuron. iTBS is posited to remove the magnesium block from NMDA receptors, facilitating calcium entry and synaptic strengthening, whereas cTBS induces a sustained depotentiation state (85).

In the context of the IHC model, the standard therapeutic application involves applying iTBS to the ipsilesional M1 to reverse diaschisis-induced hypoexcitability or applying cTBS to the contralesional M1 to dampen pathological TCI (86). Meta-analyses have consistently demonstrated that iTBS applied to the ipsilesional hemisphere significantly improves upper extremity motor scores, such as the Fugl-Meyer assessment, particularly in the subacute phase of recovery (87, 88). The mechanism is attributed to the enhancement of perilesional network excitability, which reinforces the residual corticospinal output.

However, the application of inhibitory cTBS to the contralesional hemisphere presents a significant clinical paradox that dialectically challenges the universality of the IHC model. While cTBS is theoretically sound for patients with high structural reserve (Tier 1), empirical evidence reveals distinct heterogeneity in outcomes. In patients with severe motor impairment and extensive CST damage (Tier 3), the application of cTBS to the healthy hemisphere has been observed to cause functional deterioration rather than improvement (83). This phenomenon can be explained by the Vicariation model: in the absence of a viable ipsilesional CST, the contralesional M1 assumes a compensatory role, driving motor output via uncrossed pathways or bilateral reticulospinal projections. Under these specific physiological conditions, the high excitability of the contralesional hemisphere is not a pathological source of inhibition but a necessary adaptive drive. Therefore, indiscriminately applying cTBS to the healthy side in severe stroke patients constitutes a maladaptive suppression of the patient’s only remaining functional scaffold. This contradiction highlights that the greater potency of TBS requires greater diagnostic precision; the decision to use contralesional cTBS should therefore be informed by evidence of ipsilesional CST integrity and the broader residual motor-network profile.

To resolve the conflict between the potency of TBS and the heterogeneity of stroke pathology, a stratified application algorithm is required. For patients with high structural reserve (Tier 1/2), characterized by the presence of MEPs and preserved FA in the internal capsule, a restitution protocol utilizing sequential bilateral TBS offers synergistic benefits (88). A study suggested that priming the motor cortex with cTBS to the contralesional side immediately prior to applying iTBS to the ipsilesional side can amplify the facilitatory effects of the latter (88). This priming effect is believed to function via homeostatic metaplasticity, where the prior suppression of the healthy hemisphere reduces the threshold for LTP induction in the affected hemisphere, thereby optimizing the re-balancing of interhemispheric interactions (89).

Conversely, in patients with low structural reserve (Tier 3), the physiological rationale shifts toward preserving or potentially facilitating compensatory networks. Under this interpretation, inhibitory cTBS to the contralesional hemisphere should be approached with particular caution when convergent evidence suggests dependence on contralesional motor pathways. Emerging evidence has explored facilitatory iTBS or high-frequency stimulation of the contralesional premotor cortex or M1 as potential strategies to augment compensatory drive through alternative descending pathways (90). However, the clinical evidence supporting facilitatory stimulation of the contralesional hemisphere in patients with severe stroke remains preliminary. Much of the available evidence is derived from mechanistic studies, exploratory trials, or relatively small and frequently single-center cohorts, with substantial heterogeneity in stroke chronicity, lesion characteristics, stimulation targets, and protocol parameters. In particular, the therapeutic effects of contralesional high-frequency rTMS or iTBS have not yet been established in adequately powered, multicenter Phase III randomized controlled trials. Accordingly, the physiological rationale for preserving or facilitating contralesional compensatory networks should be distinguished from definitive evidence of clinical efficacy for any specific excitatory protocol. Future biomarker-stratified trials are required to determine whether patients with low CST reserve and evidence of contralesional recruitment derive reproducible benefit from these strategies. Furthermore, a dose–response study indicates that standard 600-pulse protocols may be insufficient for resistant cases, whereas prolonged or accelerated iTBS protocols may overcome the high threshold for plasticity in chronic stroke, although the non-linear response of synaptic plasticity (where excessive stimulation might reverse the effect) requires careful monitoring (91). The integration of TBS into the clinical workflow thus demands a transition from a binary choice of excite vs. inhibit to a geometric calculation based on the specific topology of the patient’s residual motor network.

### 6.2 Cortical multi-target stimulation

The conceptual limitation of traditional rTMS lies in its reductionist focus on the M1 as the sole locus of recovery (92–94). However, the brain operates as a dynamic connectome, and ischemic injury invariably disrupts widespread functional networks extending far beyond the lesion core (95–97). Consequently, the frontier of neuromodulation is expanding toward a multi-nodal approach that targets secondary motor areas, specifically the PMd, the SMA, and the cerebellum (98–100) (Figure 3A). This topological expansion is critically important for patients with Tier 3 structural reserve, where the canonical corticospinal output from the ipsilesional M1 is functionally disconnected, rendering standard M1-focused strategies ineffective (101).

The PMd and SMA represent the pivotal nodes for vicarious recovery in severe stroke (102). Unlike the M1, which exhibits a high degree of lateralization and dependence on the crossed CST, the PMd and SMA possess extensive bilateral connectivity and serve as the primary cortical origins for the CRT (96). In the event of extensive CST ablation, the CRT provides an alternative, phylogenetically older descending pathway that can mediate proximal and postural motor control (96, 101).

Empirical studies challenging the IHC dogma have demonstrated that high-frequency rTMS applied to the contralesional PMd can facilitate functional recovery in patients with severe motor deficits (94, 103). Mechanistically, facilitating the contralesional PMd enhances the excitatory drive to the reticular formation, thereby recruiting proximal muscle groups to support hemiparetic limb movement via uncrossed pathways. These findings support the possibility that the functional contribution of the contralesional hemisphere may be region-specific: while the contralesional M1 might inhibit the ipsilesional M1 (requiring suppression in mild stroke), the contralesional PMd might drive the paretic limb (requiring excitation in severe stroke). Nevertheless, these findings are based on relatively limited clinical datasets, and the efficacy, optimal target, and stimulation parameters of contralesional PMd facilitation require confirmation in larger biomarker-stratified trials before routine clinical adoption.

Furthermore, dual-site stimulation strategies targeting the SMA offer a mechanism to reconstruct motor planning sequences. The SMA is critical for the internal generation of movement and bimanual coordination. In patients presenting with akinesia or “frozen” motor states, excitatory stimulation of the SMA, potentially in combination with M1 stimulation, has been shown to unlock motor sequences that M1 stimulation alone fails to trigger. Thus, multi-target protocols offer a “bypass strategy,” routing motor commands through secondary nodes when the primary corticospinal highway is irreversibly blocked (104).

The cerebellum constitutes a potent, yet historically underutilized, target for rTMS in stroke rehabilitation (105). Through the cerebello-thalamo-cortical (CTC) pathway, the cerebellum exerts a powerful tonic facilitatory influence on the contralateral M1. Stroke frequently induces a phenomenon known as crossed cerebellar diaschisis (CCD), where metabolic activity in the contralesional cerebellum is suppressed due to the loss of excitatory input from the infarcted hemisphere. This diaschisis contributes to the overall reduction in motor drive and impedes the relearning of complex motor patterns (106).

Stimulating the cerebellum offers a unique “backdoor” to the motor cortex. iTBS applied to the cerebellar vermis, or the lateral hemispheres can potentiate the CTC pathway, thereby enhancing excitability in the perilesional M1 via a polysynaptic route that bypasses the lesion site itself. This approach is particularly advantageous for patients with subcortical lesions where the direct cortical structure is intact but deafferented. Moreover, cerebellar stimulation has demonstrated efficacy in improving gait, balance, and fine motor coordination, domains which are often resistant to M1-only stimulation, by modulating vestibulospinal and reticulospinal outflows (98).

The synergy of combined cerebral and cerebellar stimulation represents the cutting edge of network modulation. Protocols that couple inhibitory rTMS to the contralesional M1 with facilitatory rTMS to the cerebellum have demonstrated superior efficacy compared to single-site stimulation. This dual-site approach putatively functions by simultaneously releasing the “brake” through reducing IHI from the healthy side and pressing the “accelerator” by increasing cerebellar drive to the affected side, creating a convergent facilitatory effect on the perilesional motor network. For the stratified treatment algorithm, the incorporation of cerebellar targets is essential for patients with ataxia or severe gait disturbance, acting as a functional adjunct to CST-focused strategies (107).

Beyond targeting single cortical nodes, the network perspective necessitates addressing the connectivity between regions. Cortico-cortical Paired Associative Stimulation (ccPAS) has emerged as a novel technique to modulate the efficacy of specific neural pathways based on Hebbian principles. As illustrated in Figure 3B, this protocol involves pairing stimulation over a pre-synaptic target (e.g., the ventral premotor cortex, PMv) with a post-synaptic target (M1) at a precise inter-stimulus interval. Turrini et al. demonstrated that ccPAS targeting the PMv-to-M1 pathway significantly enhances corticomotor excitability, specifically facilitating hand muscle activity involved in grasping (108). This suggests that for patients with specific deficits in hand dexterity, therapy should focus not just on exciting the M1, but on strengthening the physiological connectivity between the premotor and primary motor cortices (see Table 3).

**Figure 3:**
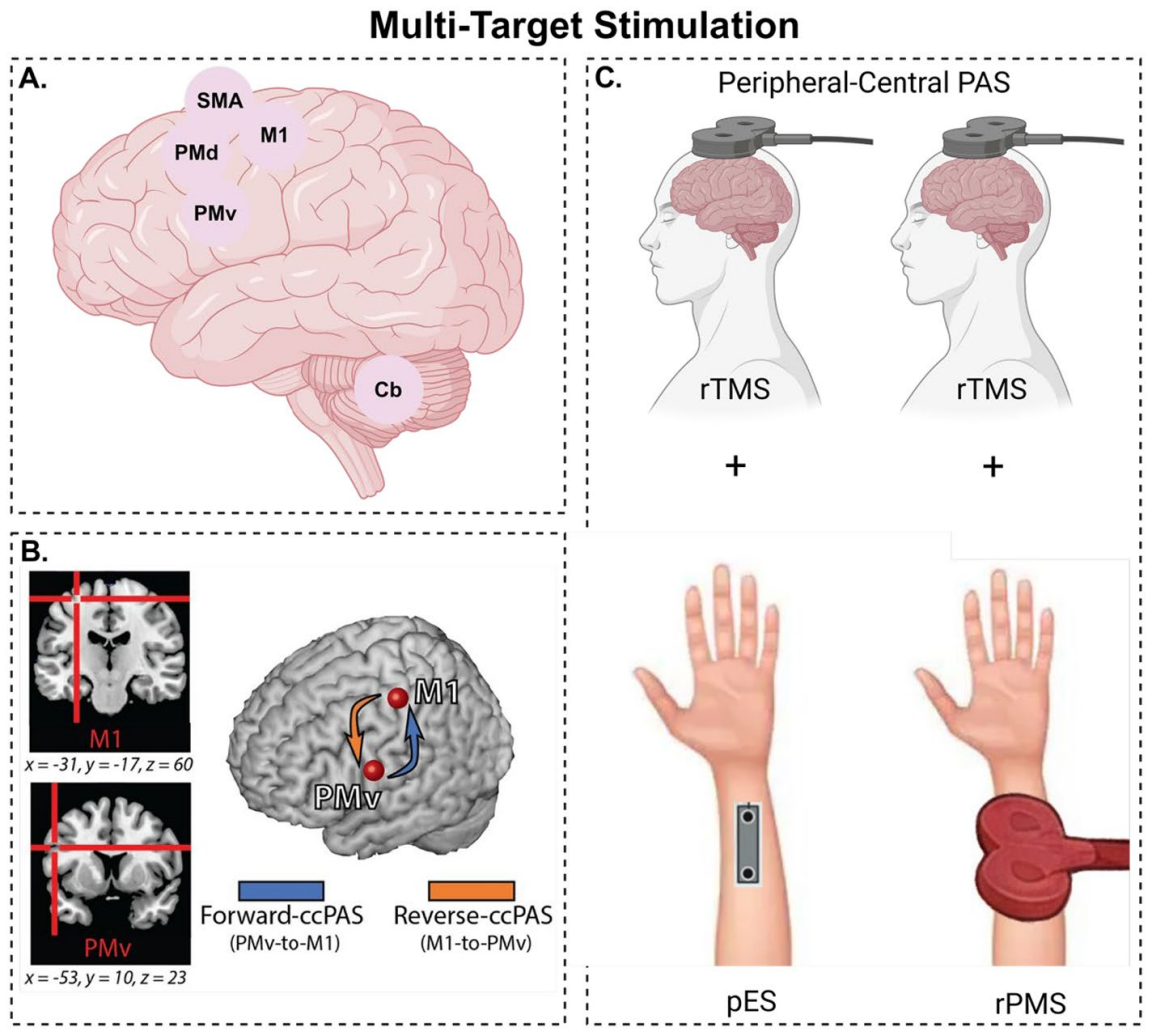
Multi-target stimulation. **(A)** Motor-related brain regions serve as potential vicarious targets (108). **(B)** Cortico-cortical paired associative stimulation (ccPAS) targeting the connection between the ventral premotor cortex (PMv) and M1 to enhance network connectivity. **(C)** Peripheral-Central combined stimulation strategies, illustrating the integration of rTMS with peripheral electrical stimulation (pES) or repetitive peripheral magnetic stimulation (rPMS) to induce Hebbian plasticity.

**Table 3:** Clinical indications and mechanisms for non-M1 stimulation targets in stroke rehabilitation.

Target region	Anatomical rationale	Clinical indication (Target Population)	rTMS strategy and mechanism	Expected functional Outcome
PMd	Origin of the CRT; possesses extensive bilateral connectivity to spinal motor pools.	Candidate population: Severe hemiparesis with extensive CST damage and evidence of compensatory network recruitment.	Investigational facilitation (high-frequency rTMS/iTBS)	Potential improvement in proximal limb control and postural stability through compensatory recruitment.
SMA	Critical node for motor planning, bimanual coordination, and internal generation of movement sequences.	Akinesia/Motor Neglect; Patients exhibiting “frozen” motor states or difficulty initiating movement sequences.	Dual-site or Focal Excitation; Reconstructs motor planning networks and unlocks sequences that M1 stimulation alone fails to trigger.	Restoration of movement initiation, sequencing, and bimanual coordination.
Cerebellum (Vermis or Lateral Hemispheres)	Modulates M1 via the CTC pathway; influences vestibulospinal/reticulospinal tracts.	Ataxia/Gait Disturbance; Patients with CCD or subcortical lesions.	Facilitation (iTBS); Potentiates the CTC pathway to release the “brake” on the perilesional M1 and modulate spinal outflows.	Enhanced gait, balance, and fine motor coordination; resolution of diaschisis.
PMv	Key node of the mirror neuron system; exerts potent effective connectivity onto M1 specific to grasping movements.	Hand Motor Deficits/Grasping Impairment; Patients requiring restoration of fine motor control and hand-object interaction.	ccPAS (PMv-to-M1); Uses STDP to strengthen the specific neural pathway from PMv to M1.	Enhanced corticomotor excitability and restoration of precision grip and hand dexterity.

CCD, crossed cerebellar diaschisis; CRT, corticoreticular tract; CST, corticospinal tract; CTC, cerebello-thalamo-cortical; iTBS, intermittent theta-burst stimulation; M1, primary motor cortex; PMd, dorsal premotor cortex; PMv, ventral premotor cortex; ccPAS, cortico-cortical paired associative stimulation; SMA, supplementary motor area. This table summarizes alternative stimulation sites for patients who are unresponsive to standard M1 protocols or exhibit specific deficits such as akinesia or ataxia. Selection of these targets should be guided by the multimodal assessment described in Section 5.

### 6.3 Peripheral-central combined stimulation

The ultimate imperative of neurorehabilitation is not merely to modulate cortical excitability in isolation but to re-integrate the brain with the peripheral effector system (109). While rTMS acts as a top-down modulator, peripheral sensory and motor inputs provide the “bottom-up” drive essential for motor learning (110). The integration of these two streams culminates in the concept of Paired Associative Stimulation (PAS) and hybrid Peripheral-Central stimulation protocols (Figure 3C). These emerging modalities leverage the principles of Spike-Timing Dependent Plasticity (STDP) and Hebbian learning, which is axiomatized as “cells that fire together, wire together,” to induce potent synaptic remodeling (111).

PAS represents the translation of cellular neuroscience into clinical practice (112). The protocol involves the precise temporal pairing of a peripheral electrical stimulus with a TMS pulse applied to the contralateral M1 (113). The inter-stimulus interval is meticulously calculated to ensure that the afferent sensory volley reaches the sensorimotor cortex exactly as the magnetic pulse depolarizes the cortical neurons (114). This coincidence detection triggers robust, long-lasting LTP-like plasticity that exceeds the effects of either modality alone (115).

In stroke survivors, traditional rehabilitation provides peripheral input while rTMS provides central input, but these inputs are rarely time-locked with the millisecond precision required for STDP (109). PAS bridges this gap by creating artificial synchrony. Emerging variants such as Visuomotor PAS, which pairs action observation with TMS, further recruit the mirror neuron system to reinforce the motor trace (116). Clinical trials utilizing repetitive peripheral magnetic stimulation (rPMS) in conjunction with rTMS have demonstrated synergistic effects, particularly in improving upper limb motor function and reducing spasticity (117). Specifically, a recent pilot study by Liang et al. confirmed that combining central iTBS with peripheral rPMS yielded superior improvements in upper extremity motor function (Fugl-Meyer Assessment) compared to central stimulation alone (111). Unlike electrical stimulation, rPMS activate deep proprioceptive fibers with minimal cutaneous pain, generating a massive sensory volley that primes the sensorimotor cortex for plasticity (118). When rTMS is applied immediately following or concurrently with this proprioceptive bombardment, the cortical circuits are placed in a state of heightened receptivity. For patients with “dormant” pathways (Tier 2/3), where voluntary movement is insufficient to drive plasticity, this artificial synchronization acts as a neuroprosthetic bridge, forcing the association between the paretic limb and the dissociated cortex (119).

The frontier of peripheral-central integration lies in the transition from open-loop to closed-loop systems (120). Traditional rTMS delivers pulses at a fixed frequency, disregarding the brain’s instantaneous physiological state (121). However, neuronal excitability is not static; it fluctuates with intrinsic oscillatory phases, particularly the sensorimotor mu-rhythm (122). Brain-state dependent stimulation utilizes real-time electroencephalography (EEG) to trigger TMS pulses only when the brain is in a specific phase of high excitability, such as the trough of the mu-oscillation (123).

This technological leap creates a biologically intelligent stimulation protocol. In a closed-loop rehabilitation setting, the system detects the patient’s motor intention via EEG event-related desynchronization (124). This intention signal triggers a peripheral exoskeleton to move the limb while providing proprioceptive feedback, and simultaneously triggers a TMS pulse to the M1. This creates a perfect, causally connected Hebbian loop consisting of the sequential steps: intention, central stimulation, peripheral action, and feedback. Early trials indicate that such state-dependent stimulation can induce plasticity with greater reliability and lower variability than standard protocols. While currently in the investigational stage, closed-loop rTMS represents the logical endpoint of precision neuromodulation, a therapy that adapts millisecond-by-millisecond to the patient’s unique neural dynamics and promises to maximize recovery outcomes even for the most refractory post-stroke deficits.

## 7 Conclusion and perspectives

The trajectory of post-stroke motor rehabilitation is moving from uniform stimulation protocols toward biomarker-informed and individualized neuromodulation. The apparent conflict between the IHC and Vicariation models may be more appropriately understood as a continuum shaped by residual motor-system architecture, including CST integrity, neurophysiological function, and the availability of compensatory networks. This review therefore proposes structural reserve as an organizing construct for integrating these complementary sources of evidence.

Within this framework, patients with relatively preserved reserve may be more consistent with an IHC-dominant profile, supporting consideration of strategies aimed at restoring ipsilesional function and interhemispheric balance. In patients with markedly depleted CST reserve and convergent evidence of contralesional compensatory recruitment, indiscriminate suppression of the contralesional hemisphere may be inappropriate, while facilitatory strategies targeting compensatory networks remain promising but require further clinical validation. Patients with discordant biomarkers constitute a dynamic gray zone in which mechanistic assignment should remain provisional and be refined through multimodal and longitudinal reassessment.

Looking forward, the integration of emerging modalities offers synergistic potential to enhance this stratified framework. TBS provides a potent, time-efficient tool that, if targeted correctly based on reserve status, can accelerate plasticity induction. Multi-target stimulation acknowledges the network nature of stroke, recruiting premotor and cerebellar nodes to bypass damaged epicenters and resolve diaschisis. Finally, closed-loop, brain-state-dependent stimulation represents the future of rehabilitation, promising to synchronize central and peripheral drives with Hebbian precision, thereby maximizing the efficiency of neural reorganization.

In conclusion, the clinical efficacy of rTMS is not limited by the technology itself but by the precision of its application. By adopting a dialectical approach that reconciles competing models through objective biomarkers, such frameworks may reduce reliance on empirical trial-and-error selection. Future research must focus on validating these stratified algorithms in large-scale, multi-center trials and developing accessible point-of-care biomarkers to democratize precision neuromodulation. The era of generic brain stimulation is ending; the era of individualized, anatomically-informed, and network-targeted neurorehabilitation has begun.

## Glossary

CCD: Crossed Cerebellar Diaschisis
ccPAS: Cortico-cortical Paired Associative Stimulation
CRT: Corticoreticular Tract
CST: Corticospinal Tract
CTC: Cerebello-Thalamo-Cortical
cTBS: Continuous Theta-Burst Stimulation
DCM: Dynamic Causal Modeling
DTI: Diffusion Tensor Imaging
EEG: Electroencephalography
FA: Fractional Anisotropy
FA_asym: Fractional Anisotropy Asymmetry Index
HARDI: High-Angular Resolution Diffusion Imaging
IHC: Interhemispheric Competition
IHI: Interhemispheric Inhibition
iMEP: Ipsilateral Motor Evoked Potential
iSP: Ipsilateral Silent Period
iTBS: Intermittent Theta-Burst Stimulation
LTD: Long-Term Depression
LTP: Long-Term Potentiation
M1: Primary Motor Cortex
MD: Mean Diffusivity
MEP: Motor Evoked Potential
NMDA: N-methyl-D-aspartate
PAS: Paired Associative Stimulation
pES: Peripheral Electrical Stimulation
PLIC: Posterior Limb of the Internal Capsule
PMC: Premotor Cortex
PMd: Dorsal Premotor Cortex
PMv: Ventral Premotor Cortex
PREP2: Predict Recovery Potential 2
RD: Radial Diffusivity
rFA: Ratio Fractional Anisotropy
rPMS: Repetitive Peripheral Magnetic Stimulation
RST: Reticulospinal Tract
rTMS: Repetitive Transcranial Magnetic Stimulation
SAFE: Shoulder Abduction and Finger Extension
SMA: Supplementary Motor Area
SPiRe: Stroke Precision in Reservoir-guided Neuromodulation
START: Stratification Algorithm for Repetitive TMS in Stroke
STDP: Spike-Timing Dependent Plasticity
TBS: Theta-Burst Stimulation
TCI: Transcallosal Inhibition
tDCS: Transcranial Direct Current Stimulation
TI: Temporal Interference
TUS: Transcranial Ultrasound Stimulation

## References

[1] GBD 2019 Stroke Collaborators. Global, regional, and national burden of stroke and its risk factors, 1990-2019: a systematic analysis for the global burden of disease study 2019. Lancet Neurol. (2021) 20:795–820. doi: 10.1016/S1474-4422(21)00252-0, 34487721 PMC8443449

[2] Murphy TH Corbett D. Plasticity during stroke recovery: from synapse to behaviour. Nat Rev Neurosci. (2009) 10:861–72. doi: 10.1038/nrn2735 19888284

[3] Kwakkel G Kollen B Lindeman E. Understanding the pattern of functional recovery after stroke: facts and theories. Restor Neurol Neurosci (2004) 22:281–99. doi: 10.3233/RNN-2004-00282 15502272

[4] Campbell BCV De Silva DA Macleod MR Coutts SB Schwamm LH Davis SM . Ischaemic stroke. Nat Rev Dis Primers. (2019) 5:70. doi: 10.1038/s41572-019-0118-8 31601801

[5] Qian Q Nam C Guo Z Huang Y Hu X Ng SC . Distal versus proximal - an investigation on different supportive strategies by robots for upper limb rehabilitation after stroke: a randomized controlled trial. J Neuroeng Rehabil. (2019) 16:64. doi: 10.1186/s12984-019-0537-5 PMC6545723 31159822

[6] Langhorne P Bernhardt J Kwakkel G. Stroke rehabilitation. Lancet (London, England). (2011) 377:1693–702. doi: 10.1016/S0140-6736(11)60325-5 21571152

[7] Ward NS. Restoring brain function after stroke - bridging the gap between animals and humans. Nat Rev Neurol. (2017) 13:244–55. doi: 10.1038/nrneurol.2017.34 28303914

[8] Lefaucheur JP André-Obadia N Antal A Ayache SS Baeken C Benninger DH . Evidence-based guidelines on the therapeutic use of repetitive transcranial magnetic stimulation (rTMS). Clin Neurophysiol. (2014) 125:2150–206. doi: 10.1016/j.clinph.2014.05.021 25034472

[9] Kandel M Beis JM Le Chapelain L Guesdon H Paysant J. Non-invasive cerebral stimulation for the upper limb rehabilitation after stroke: a review. Ann Phys Rehabil Med. (2012) 55:657–680. doi: 10.1016/j.rehab.2012.09.001 23084320

[10] Bliss TVP Collingridge GL. A synaptic model of memory: long-term potentiation in the hippocampus. Nature. (1993) 361:31–9. doi: 10.1038/361031a0 8421494

[11] Huang YZ Edwards MJ Rounis E Bhatia KP Rothwell JC. Theta burst stimulation of the human motor cortex. Neuron. (2005) 45:201–6. doi: 10.1016/j.neuron.2004.12.033 15664172

[12] Alhalabi H Qiu Y Wei Y Chen Y Gao Q. Optimizing repetitive transcranial magnetic stimulation (rTMS) protocols for upper limb motor recovery in post-stroke patients: a meta-analysis. Restor Neurol Neurosci. (2025) 43:122–37. doi: 10.1177/09226028251394303 PMC13328933 41329858

[13] Fisicaro F Lanza G Grasso AA Pennisi G Bella R Paulus W . Repetitive transcranial magnetic stimulation in stroke rehabilitation: review of the current evidence and pitfalls. Ther Adv Neurol Disord. (2019) 12:1756286419878317. doi: 10.1177/1756286419878317 PMC6763938 31598137

[14] Grefkes C Ward NS. Cortical reorganization after stroke: how much and how functional? Neuroscientist. (2014) 20:56–70. doi: 10.1177/1073858413491147 23774218

[15] Hummel FC Cohen LG. Non-invasive brain stimulation: a new strategy to improve neurorehabilitation after stroke? Lancet Neurol. (2006) 5:708–12. doi: 10.1016/S1474-4422(06)70525-7, 16857577

[16] Murase N Duque J Mazzocchio R Cohen LG. Influence of interhemispheric interactions on motor function in chronic stroke. Ann Neurol. (2004) 55:400–9. doi: 10.1002/ana.10848 14991818

[17] Nowak DA Grefkes C Ameli M Fink GR. Interhemispheric competition after stroke: brain stimulation to enhance recovery of function of the affected hand. Neurorehabil Neural Repair. (2009) 23:641–56. doi: 10.1177/1545968309336661 19531606

[18] Takeuchi N Izumi S-I. Noninvasive brain stimulation for motor recovery after stroke: mechanisms and future views. Stroke Res Treat. (2012) 2012:584727. doi: 10.1155/2012/584727 PMC3463193 23050198

[19] Grefkes C Nowak DA Eickhoff SB Dafotakis M Küst J Karbe H . Cortical connectivity after subcortical stroke assessed with functional magnetic resonance imaging. Ann Neurol. (2008) 63:236–46. doi: 10.1002/ana.21228 17896791

[20] Di Pino G Pellegrino G Assenza G Capone F Ferreri F Formica D . Modulation of brain plasticity in stroke: a novel model for neurorehabilitation. Nat Rev Neurol. (2014) 10:597–608. doi: 10.1038/nrneurol.2014.162 25201238

[21] Stoeckel MC Binkofski F. The role of ipsilateral primary motor cortex in movement control and recovery from brain damage. Exp Neurol. (2010) 221:13–7. doi: 10.1016/j.expneurol.2009.10.021, 19896482

[22] Stagg CJ Nitsche MA. Physiological basis of transcranial direct current stimulation. Neuroscientist. (2011) 17:37–53. doi: 10.1177/1073858410386614 21343407

[23] Lefaucheur JP Aleman A Baeken C Benninger DH Brunelin J Di Lazzaro V . Evidence-based guidelines on the therapeutic use of repetitive transcranial magnetic stimulation (rTMS): an update (2014-2018). Clin Neurophysiol. (2020) 131:474–528. doi: 10.1016/j.clinph.2019.11.002 31901449

[24] Grossman N Bono D Dedic N Kodandaramaiah SB Rudenko A Suk HJ . Noninvasive deep brain stimulation via temporally interfering electric fields. Cell. (2017) 169:1029–1041.e16. doi: 10.1016/j.cell.2017.05.024 PMC5520675 28575667

[25] Legon W Sato TF Opitz A Mueller J Barbour A Williams A . Transcranial focused ultrasound modulates the activity of primary somatosensory cortex in humans. Nat Neurosci. (2014) 17:322–29. doi: 10.1038/nn.3620 24413698

[26] Hallett M. Transcranial magnetic stimulation and the human brain. Nature. (2000) 406:147–50. doi: 10.1038/35018000 10910346

[27] Rossini PM Burke D Chen R Cohen LG Daskalakis Z Di Iorio R . Non-invasive electrical and magnetic stimulation of the brain, spinal cord, roots and peripheral nerves: basic principles and procedures for routine clinical and research application. An updated report from an I.F.C.N. Committee. Clin Neurophysiol. (2015) 126:1071–107. doi: 10.1016/j.clinph.2015.02.001 PMC6350257 25797650

[28] Suppa A Huang YZ Funke K Ridding MC Cheeran B Di Lazzaro V . Ten years of theta burst stimulation in humans: established knowledge, unknowns and prospects. Brain Stimul. (2016) 9:323–35. doi: 10.1016/j.brs.2016.01.006 26947241

[29] Stinear CM. Prediction of motor recovery after stroke: advances in biomarkers. Lancet Neurol. (2017) 16:826–36. doi: 10.1016/S1474-4422(17)30283-1, 28920888

[30] Ferbert A Priori A Rothwell JC Day BL Colebatch JG Marsden CD. Interhemispheric inhibition of the human motor cortex. J Physiol. (1992) 453:525–46. doi: 10.1113/jphysiol.1992.sp019243 PMC1175572 1464843

[31] Di Lazzaro V Oliviero A Profice P Insola A Mazzone P Tonali P . Direct demonstration of interhemispheric inhibition of the human motor cortex produced by transcranial magnetic stimulation. Exp Brain Res. (1999) 124:520–24. doi: 10.1007/s002210050648 10090664

[32] Gerges ANH Hordacre B Di Pietro F Moseley GL Berryman C. Do adults with stroke have altered interhemispheric inhibition? A systematic review with meta-analysis. J Stroke Cerebrovasc Dis Off J Natl Stroke Assoc. (2022) 31:106494. doi: 10.1016/j.jstrokecerebrovasdis.2022.106494 35472653

[33] Boddington LJ Reynolds JNJ. Targeting interhemispheric inhibition with neuromodulation to enhance stroke rehabilitation. Brain Stimul. (2017) 10:214–22. doi: 10.1016/j.brs.2017.01.006 28117178

[34] Ward NS Brown MM Thompson AJ Frackowiak RSJ. Neural correlates of outcome after stroke: a cross-sectional fMRI study. Brain J Neurol. (2003) 126:1430–48. doi: 10.1093/brain/awg145, 12764063 PMC3717456

[35] Marshall RS Perera GM Lazar RM Krakauer JW Constantine RC DeLaPaz RL. Evolution of cortical activation during recovery from corticospinal tract infarction. Stroke. (2000) 31:656–61. doi: 10.1161/01.str.31.3.656, 10700500

[36] Carey JR Kimberley TJ Lewis SM Auerbach EJ Dorsey L Rundquist P . Analysis of fMRI and finger tracking training in subjects with chronic stroke. Brain. (2002) 125:773–88. doi: 10.1093/brain/awf091 11912111

[37] Shen Y Du J Yao X Tang J. A meta-analysis of the effects of transcranial magnetic stimulation on hand function and daily living ability after stroke. Medicine. (2025) 104:e44029. doi: 10.1097/MD.0000000000044029 PMC12401453 40898556

[38] Li R Liu S Li T Yang K Wang X Wang W. The stratified effects of repetitive transcranial magnetic stimulation in upper limb motor impairment recovery after stroke: a meta-analysis. Front Neurol. (2024) 15:1369836. doi: 10.3389/fneur.2024.1369836 PMC11020108 38628695

[39] Graziadio S Tomasevic L Assenza G Tecchio F Eyre JA. The myth of the ‘unaffected’ side after unilateral stroke: is reorganisation of the non-infarcted corticospinal system to re-establish balance the price for recovery? Exp Neurol. (2012) 238:168–75. doi: 10.1016/j.expneurol.2012.08.031, 22981842 PMC3508413

[40] Brancaccio A Tabarelli D Belardinelli P. A new framework to interpret individual inter-hemispheric compensatory communication after stroke. J Pers Med. (2022) 12:59. doi: 10.3390/jpm12010059 PMC8778334 35055374

[41] Carmichael ST. Plasticity of cortical projections after stroke. Neuroscientist. (2003) 9:64–75. doi: 10.1177/1073858402239592 12580341

[42] Johansen-Berg H Rushworth MFS Bogdanovic MD Kischka U Wimalaratna S Matthews PM. The role of ipsilateral premotor cortex in hand movement after stroke. Proc Natl Acad Sci USA. (2002) 99:14518–23. doi: 10.1073/pnas.222536799 PMC137915 12376621

[43] Ramos-Murguialday A García-Cossio E Walter A Cho W Broetz D Bogdan M . Decoding upper limb residual muscle activity in severe chronic stroke. Ann Clin Transl Neurol. (2015) 2:1–11. doi: 10.1002/acn3.122, 25642429 PMC4301668

[44] Jang SH Cho MJ. Role of the contra-lesional corticoreticular tract in motor recovery of the paretic leg in stroke: a mini-narrative review. Front Hum Neurosci. (2022) 16:896367. doi: 10.3389/fnhum.2022.896367 PMC9204517 35721363

[45] Oquita R Cuello V Uppati S Mannuru S Salinas D Dobbs M . Moving toward elucidating alternative motor pathway structures post-stroke: the value of spinal cord neuroimaging. Front Neurol. (2024) 15:1282685. doi: 10.3389/fneur.2024.1282685 PMC10899520 38419695

[46] Schulz R Park E Lee J Chang WH Lee A Kim YH . Interactions between the corticospinal tract and premotor-motor pathways for residual motor output after stroke. Stroke. (2017) 48:2805–11. doi: 10.1161/STROKEAHA.117.016834 28904231

[47] Sankarasubramanian V Machado AG Conforto AB Potter-Baker KA Cunningham DA Varnerin NM . Inhibition versus facilitation of contralesional motor cortices in stroke: deriving a model to tailor brain stimulation. Clin Neurophysiol. (2017) 128: 892–902. doi: 10.1016/j.clinph.2017.03.030 PMC5493996 28402865

[48] Byblow WD Stinear CM Barber PA Petoe MA Ackerley SJ. Proportional recovery after stroke depends on corticomotor integrity. Ann Neurol. (2015) 78:848–59. doi: 10.1002/ana.24472, 26150318

[49] Bradnam LV Stinear CM Byblow WD. Ipsilateral motor pathways after stroke: implications for non-invasive brain stimulation. Front Hum Neurosci. (2013) 7:184. doi: 10.3389/fnhum.2013.00184 PMC3647244 23658541

[50] Li S Chen YT Francisco GE Zhou P Rymer WZ. A unifying pathophysiological account for post-stroke spasticity and disordered motor control. Front Neurol. (2019) 10:468. doi: 10.3389/fneur.2019.00468, 31133971 PMC6524557

[51] Bestmann S Swayne O Blankenburg F Ruff CC Teo J Weiskopf N . The role of contralesional dorsal premotor cortex after stroke as studied with concurrent TMS-fMRI. J Neurosci. (2010) 30:11926–37. doi: 10.1523/JNEUROSCI.5642-09.2010 PMC3044467 20826657

[52] Grefkes C Fink GR. Reorganization of cerebral networks after stroke: new insights from neuroimaging with connectivity approaches. Brain. (2011) 134:1264–76. doi: 10.1093/brain/awr033 PMC3097886 21414995

[53] Volz LJ Vollmer M Michely J Fink GR Rothwell JC Grefkes C. Time-dependent functional role of the contralesional motor cortex after stroke. Neuroimage Clin. (2017) 16:165–74. doi: 10.1016/j.nicl.2017.07.024 PMC5540833 28794977

[54] Stinear C. Prediction of recovery of motor function after stroke. Lancet Neurol. (2010) 9:1228–32. doi: 10.1016/S1474-4422(10)70247-7 21035399

[55] Bertolucci F Chisari C Fregni F. The potential dual role of transcallosal inhibition in post-stroke motor recovery. Restor Neurol Neurosci. (2018) 36:83–97. doi: 10.3233/RNN-170778 29439366

[56] Kobayashi M Hutchinson S Théoret H Schlaug G Pascual-Leone A. Repetitive TMS of the motor cortex improves ipsilateral sequential simple finger movements. Neurology. (2004) 62:91–8. doi: 10.1212/wnl.62.1.91 14718704

[57] Ward NS Brown MM Thompson AJ Frackowiak RS. Neural correlates of motor recovery after stroke: a longitudinal fMRI study. Brain J Neurol. (2003) 126:2476–96. doi: 10.1093/brain/awg245 PMC3717457 12937084

[58] Yoo YJ Kim JW Kim JS Hong BY Lee KB Lim SH. Corticospinal tract integrity and long-term hand function prognosis in patients with stroke. Front Neurol. (2019) 10:374. doi: 10.3389/fneur.2019.00374 PMC6476282 31037066

[59] Bradnam LV Stinear CM Barber PA Byblow WD. Contralesional hemisphere control of the proximal paretic upper limb following stroke. Cereb Cortex. (2012) 22:2662–71. doi: 10.1093/cercor/bhr344 PMC4705341 22139791

[60] Shimizu T Hosaki A Hino T Sato M Komori T Hirai S . Motor cortical disinhibition in the unaffected hemisphere after unilateral cortical stroke. Brain. (2002) 125:1896–907. doi: 10.1093/brain/awf183 12135979

[61] Buetefisch CM. Role of the Contralesional hemisphere in post-stroke recovery of upper extremity motor function. Front Neurol. (2015) 6:214. doi: 10.3389/fneur.2015.00214, 26528236 PMC4607877

[62] Stinear CM Byblow WD Ackerley SJ Smith MC Borges VM Barber PA. PREP2: A biomarker-based algorithm for predicting upper limb function after stroke. Ann Clin Transl Neurol. (2017) 4:811–20. doi: 10.1002/acn3.488, 29159193 PMC5682112

[63] Beaulieu C. The basis of anisotropic water diffusion in the nervous system - a technical review. NMR Biomed. (2002) 15:435–55. doi: 10.1002/nbm.782, 12489094

[64] Stinear CM Barber PA Petoe M Anwar S Byblow WD. The PREP algorithm predicts potential for upper limb recovery after stroke. Brain. (2012) 135:2527–35. doi: 10.1093/brain/aws146, 22689909

[65] Puig J Pedraza S Blasco G Daunis-i-Estadella J Prats A Prados F . Wallerian degeneration in the corticospinal tract evaluated by diffusion tensor imaging correlates with motor deficit 30 days after middle cerebral artery ischemic stroke. AJNR Am J Neuroradiol. (2010) 31:1324–30. doi: 10.3174/ajnr.A2038, 20299434 PMC7965455

[66] Lindenberg R Renga V Zhu LL Betzler F Alsop D Schlaug G. Structural integrity of corticospinal motor fibers predicts motor impairment in chronic stroke. Neurology. (2010) 74:280–7. doi: 10.1212/WNL.0b013e3181ccc6d9, 20101033 PMC3122304

[67] Baker SN. The primate reticulospinal tract, hand function and functional recovery. J Physiol. (2011) 589:5603–12. doi: 10.1113/jphysiol.2011.215160, 21878519 PMC3249036

[68] Thomalla G Glauche V Koch MA Beaulieu C Weiller C Röther J. Diffusion tensor imaging detects early Wallerian degeneration of the pyramidal tract after ischemic stroke. NeuroImage. (2004) 22:1767–74. doi: 10.1016/j.neuroimage.2004.03.041, 15275932

[69] Puig J Blasco G Daunis-I-Estadella J Thomalla G Castellanos M Figueras J . Decreased corticospinal tract fractional anisotropy predicts long-term motor outcome after stroke. Stroke. (2013) 44:2016–8. doi: 10.1161/STROKEAHA.111.000382, 23652266

[70] Alagona G Delvaux V Gérard P De Pasqua V Pennisi G Delwaide PJ . Ipsilateral motor responses to focal transcranial magnetic stimulation in healthy subjects and acute-stroke patients. Stroke. (2001) 32:1304–9. doi: 10.1161/01.str.32.6.1304, 11387491

[71] Yoo YJ Jun SY Park EJ Kim Y. Accuracy of measurements of the structural reserve for upper limb function in patients with moderate-to-severe stroke. Stroke. (2023) 54:1854–1862. doi: 10.1161/STROKEAHA.123.043109 37272392

[72] Millot S Daghsen L Checkouri T Wittwer A Valabregue R Galanaud D . Prediction of upper limb motor recovery by the PREP2 algorithm in a nonselected population: external validation and influence of cognitive syndromes. Neurorehabil Neural Repair. (2024) 38:764–74. doi: 10.1177/15459683241270056, 39162251

[73] Lundquist CB Nielsen JF Arguissain FG Brunner IC. Accuracy of the upper limb prediction algorithm PREP2 applied 2 weeks poststroke: a prospective longitudinal study. Neurorehabil Neural Repair Published online November 20, 2020. (2021) 35:68–78. doi: 10.1177/1545968320971763, 33218284

[74] Lin YL Potter-Baker KA Sankarasubramanian V Cunningham DA Li M O'Laughlin K . Stratification algorithm for repetitive TMS in stroke (START): results from an exploratory crossover study. J Neurol Sci. (2025) 473:123478. doi: 10.1016/j.jns.2025.123478, 40209285 PMC13633852

[75] Luk KY Ouyang HX Pang MYC. Low-frequency rTMS over Contralesional M1 increases Ipsilesional cortical excitability and motor function with decreased interhemispheric asymmetry in subacute stroke: A randomized controlled study. Neural Plast. (2022) 3815357:1–13. doi: 10.1155/2022/3815357, 35035473 PMC8756161

[76] Saltão da Silva MA Baune NA Belagaje S Borich MR. Clinical imaging-derived metrics of corticospinal tract structural integrity are associated with post-stroke motor outcomes: A retrospective study. Front Neurol. (2022) 13:804133. doi: 10.3389/fneur.2022.804133, 35250812 PMC8893034

[77] Vora I McKiernan S Hochberg L Kimberley T Lin D. Examining the relationship between corticospinal tract anatomy, physiology, and upper extremity motor function after acute stroke. Brain Stim. (2023) 16:369. doi: 10.1016/j.brs.2023.01.725

[78] Attia M McCarthy D Abdelghani M. Repetitive transcranial magnetic stimulation for treating chronic neuropathic pain: a systematic review. Curr Pain Headache Rep. (2021) 25:48. doi: 10.1007/s11916-021-00960-5, 33978846

[79] Sui Y Zhang JJ Fong KNK. Task-based fMRI assessment of rTMS over the primary motor cortex in poststroke hemiparetic upper extremity rehabilitation: A systematic review. J Neurol Sci. (2025) 476:123623. doi: 10.1016/j.jns.2025.123623, 40729843

[80] Corti M Patten C Triggs W. Repetitive transcranial magnetic stimulation of motor cortex after stroke: a focused review. Am J Phys Med Rehabil. (2012) 91:254–70. doi: 10.1097/PHM.0b013e318228bf0c, 22042336

[81] Bashir S Shafi H Siddiqi FA. Prediction of post stroke recovery: artificial intelligence could be a key of success. J Pak Med Assoc. (2022) 72:1677. doi: 10.47391/JPMA.5058, 36280949

[82] Fu Y Wang C Zhang L Ji D Xiang A Qi J . The effectiveness of theta burst stimulation for motor recovery after stroke: a systematic review. Eur J Med Res. (2024) 29:568. doi: 10.1186/s40001-024-02170-2, 39609900 PMC11605871

[83] Ackerley SJ Stinear CM Barber PA Byblow WD. Combining theta burst stimulation with training after subcortical stroke. Stroke. (2010) 41:1568–72. doi: 10.1161/STROKEAHA.110.583278, 20489170

[84] Liu M Wan C Wang C Li X. Effects of intermittent theta burst stimulation on upper limb motor recovery in early stroke patients: an fNIRS study. Front Neurol. (2025) 16:1542827. doi: 10.3389/fneur.2025.1542827, 40078177 PMC11896818

[85] Chen K Sun M Zhuang H. Effect of theta burst stimulation on lower extremity motor function improvement and balance recovery in patients with stroke: A systematic review and meta-analysis of randomized controlled trials. Medicine (Baltimore). (2024) 103:e40098. doi: 10.1097/MD.0000000000040098, 39495989 PMC11537599

[86] Tang Z Huang J Zhou Y Ren J Duan X Fu X . Efficacy and safety of high-dose TBS on poststroke upper extremity motor impairment: a randomized controlled trial. Stroke. (2024) 55:2212–2220. doi: 10.1161/STROKEAHA.124.046597 PMC11346718 39016009

[87] Lu J Huang J Ye A Xie C Bu P Kang J . Effect of intermittent theta burst stimulation on upper limb function in stroke patients: a systematic review and meta-analysis. Front Neurol. (2024) 15:1450435. doi: 10.3389/fneur.2024.1450435, 39463790 PMC11505115

[88] Zhang JJ Fong KNK. Effects of priming intermittent theta burst stimulation on upper limb motor recovery after stroke: study protocol for a proof-of-concept randomised controlled trial. BMJ Open. (2020) 10:e035348. doi: 10.1136/bmjopen-2019-035348, 32152174 PMC7064082

[89] Jiang S Han T Zhang Z Wen M Li Y. Effects of central intermittent theta-burst stimulation combined with repetitive peripheral magnetic stimulation on upper limb function in stroke patients. Colombia Med. (2023) 54:e2005766. doi: 10.25100/cm.v54i4.5766, 39184958 PMC11341120

[90] Dai W Yang X Liu C Ding H Guo C Zhu Y . Effects of repetitive transcranial magnetic stimulation over the contralesional dorsal premotor cortex on upper limb function in severe ischaemic stroke: study protocol for a randomised controlled trial. BMJ Open. (2023) 13:e074037. doi: 10.1136/bmjopen-2023-074037, 38070912 PMC10729250

[91] Gao B Wang Y Zhang D Wang Z Wang Z. Intermittent theta-burst stimulation with physical exercise improves poststroke motor function: a systemic review and meta-analysis. Front Neurol. (2022) 13:964627. doi: 10.3389/fneur.2022.964627, 36110393 PMC9468864

[92] Zhou B Qi J Chen K Li H Liu J Zhou Q . Localizing function-specific targets for transcranial magnetic stimulation in the absence of navigation equipment. J Visual Exp. (2025) 219:e67888. doi: 10.3791/67888, 40489407

[93] Beynel L Powers JP Appelbaum LG. Effects of repetitive transcranial magnetic stimulation on resting-state connectivity: a systematic review. NeuroImage. (2020) 211:116596. doi: 10.1016/j.neuroimage.2020.116596, 32014552 PMC7571509

[94] Tomeh A Yusof Khan AHK Wan Sulaiman WA. Repetitive transcranial magnetic stimulation of the primary motor cortex in stroke survivors-more than motor rehabilitation: a mini-review. Front Aging Neurosci. (2022) 14:897837. doi: 10.3389/fnagi.2022.897837, 36225893 PMC9549351

[95] Mustin M Hensel L Fink GR Grefkes C Tscherpel C. Individual contralesional recruitment in the context of structural reserve in early motor reorganization after stroke. NeuroImage. (2024) 300:120828. doi: 10.1016/j.neuroimage.2024.120828, 39293355

[96] van Assche M Dirren E Bourgeois A Kleinschmidt A Richiardi J Carrera E. Periinfarct rewiring supports recovery after primary motor cortex stroke. J Cereb Blood Flow Metab. (2021) 41:2174–84. doi: 10.1177/0271678X211002968, 33757315 PMC8392854

[97] Wiemer VM Paul T Hensel L Cieslak M Tscherpel C Grefkes C . Structural reserve-dependent cortical rerouting of motor control after stroke. Brain. (2026) 149:2505–15. doi: 10.1093/brain/awaf434, 41235408 PMC13337246

[98] Kilic U Smets C Deng Z Boogers A Mc Laughlin M de Vloo P . A narrative review on the current landscape of invasive neuromodulation for Poststroke motor recovery: mechanisms, challenges, and future directions. Neuromodulation. (2025) 28:1093–103. doi: 10.1016/j.neurom.2025.06.004, 40684383

[99] Simonetta C Conti M Bissacco J Ferrari V Salimei C Carparelli F . Anodal M1 tDCS shapes frequency-specific functional connectivity and network topology in Parkinson’s disease. Brain Stimul. (2025) 18:1966–77. doi: 10.1016/j.brs.2025.10.016 41151703

[100] Xu YW Lin P Yao PS Zheng SF Kang DZ. Structure and function of corticospinal projection originating from supplementary motor area. Neuroradiology. (2021) 63:1283–92. doi: 10.1007/s00234-021-02669-z, 33611621

[101] Urbin MA Liu F Moon CH. Microstructural integrity within the damaged region of the residual corticofugal projection from primary motor cortex predicts the effect of noninvasive neuromodulation targeting the spinal cord in chronic stroke. Neurotherapeutics. (2025) 22:e00607. doi: 10.1016/j.neurot.2025.e00607, 40383664 PMC12418405

[102] Ben Dor N Raffa G Scibilia A Espahbodinea S Garcia C La Torre D . Motor network pre-habilitation by low-frequency repetitive transcranial magnetic stimulation. A proof-of-concept. Acta Neurochir. (2025) 167:182. doi: 10.1007/s00701-025-06592-7 PMC12214039 40591056

[103] McCambridge AB Stinear JW Byblow WD. Revisiting interhemispheric imbalance in chronic stroke: a tDCS study. Clin Neurophysiol. (2018) 129:42–50. doi: 10.1016/j.clinph.2017.10.016, 29145166

[104] Ilmoniemi RJ Granö I Kičić D Levola J Paunio T Roine T . A paradigm shift in neuromodulation: the promise of personalized multi-locus TMS for targeted treatment of neuropsychiatric conditions. Brain Stimul. (2025) 18:303. doi: 10.1016/j.brs.2024.12.264

[105] Xu Q Zhang Z Zhai Y Chen J Xu J Chen H . Transcranial magnetic stimulation through attenuating blood-spinal cord barrier disruption and reducing inflammatory response to improve motor function in rats with spinal cord injury. Brain Res Bull. (2025) 226:111385. doi: 10.1016/j.brainresbull.2025.111385, 40367765

[106] Wang T Guo M Wu H Liu S Ji Y Wang P . rTMS modulates early AD progression via synergistic brain network reorganization and peripheral biomarker dynamics. GeroScience. (2026) 48:4295–311. doi: 10.1007/s11357-025-01888-z, 41015617 PMC13356243

[107] França C de Andrade DC Teixeira MJ Galhardoni R Silva V Barbosa ER . Effects of cerebellar neuromodulation in movement disorders: a systematic review. Brain Stimul. (2018) 11:249–60. doi: 10.1016/j.brs.2017.11.015 29191439

[108] Turrini S Fiori F Chiappini E Santarnecchi E Romei V Avenanti A. Gradual enhancement of corticomotor excitability during cortico-cortical paired associative stimulation. Sci Rep. (2022) 12:14670. doi: 10.1038/s41598-022-18774-9, 36038605 PMC9424198

[109] Martino Cinnera A Spampinato DA Pezzopane V Antonioni A Fregna G Baroni A . Promoting spike-timing-dependent plasticity via paired associative stimulation: from healthy subjects to clinical applications. Neurosci Biobehav Rev. (2025) 177:106314. doi: 10.1016/j.neubiorev.2025.106314, 40754037

[110] Luo S Wen Z Liu Y Sun T Xu L Yu Q. Effects of repetitive transcranial magnetic stimulation combined with repetitive peripheral magnetic stimulation on upper limb motor function after stroke: a systematic review and meta-analysis. Front Neurol. (2024) 15:1472837. doi: 10.3389/fneur.2024.1472837, 39600429 PMC11588637

[111] Liang S Wang W Yu F Pan L Xu D Hu R . Repetitive peripheral magnetic stimulation combined with transcranial magnetic stimulation in rehabilitation of upper extremity hemiparesis following stroke: a pilot study. J Rehabil Med. (2024) 56:jrm19449. doi: 10.2340/jrm.v56.19449, 38298134 PMC10847975

[112] Qin Y Liu X Zhang Y Wu J Wang X. Effects of transcranial combined with peripheral repetitive magnetic stimulation on limb spasticity and resting-state brain activity in stroke patients. Front Hum Neurosci. (2023) 17:992424. doi: 10.3389/fnhum.2023.992424, 37082150 PMC10110929

[113] Baker SN Zaaimi B. Boosting motor cortex plasticity through respiratory phase-triggered paired associative stimulation. bioRxiv [Preprint]. (2025). doi: 10.1101/2025.01.29.635613

[114] Zhang JP Xing XX Zheng MX Wu JJ Xue X Li YL . Effects of cortico-cortical paired associative stimulation based on multisensory integration to brain network connectivity in stroke patients: study protocol for a randomized doubled blind clinical trial. BMC Neurol. (2023) 23:176. doi: 10.1186/s12883-023-03218-2, 37118658 PMC10148448

[115] Baroni A Antonioni A Fregna G Lamberti N Manfredini F Koch G . The effectiveness of paired associative stimulation on motor recovery after stroke: a scoping review. Neurol Int. (2024) 16:567–89. doi: 10.3390/neurolint16030043, 38804482 PMC11130975

[116] Bevacqua N Turrini S Fiori F Saracini C Lucero B Candidi M . Cortico-cortical paired associative stimulation highlights asymmetrical communication between rostral premotor cortices and primary motor cortex. Brain Stimul. (2024) 17:89–91. doi: 10.1016/j.brs.2024.01.001, 38191092

[117] Wang Y Fong KNK Sui Y Bai Z Zhang JJ. Repetitive peripheral magnetic stimulation alone or in combination with repetitive transcranial magnetic stimulation in poststroke rehabilitation: a systematic review and meta-analysis. J Neuroeng Rehabil. (2024) 21:181. doi: 10.1186/s12984-024-01486-8, 39407278 PMC11481378

[118] Liao D He Z Yan S Ji Q Li Y Tu Y . Efficacy of repeated peripheral magnetic stimulation on upper limb motor function after stroke: a systematic review and meta-analysis of randomized controlled trials. Front Neurol. (2025) 16:1513826. doi: 10.3389/fneur.2025.1513826, 40248011 PMC12003123

[119] Yamamoto S Aoyama T Ishii D Ishibashi K Kohno Y. Case report: repetitive peripheral magnetic stimulation and task-oriented training improve motor function in chronic severe post-stroke paralysis. Front Stroke. (2025) 4:1547280. doi: 10.3389/fstro.2025.1547280, 41541876 PMC12802746

[120] Zrenner C Desideri D Belardinelli P Ziemann U. Real-time EEG-defined excitability states determine efficacy of TMS-induced plasticity in human motor cortex. Brain Stimul. (2018) 11:374–89. doi: 10.1016/j.brs.2017.11.016, 29191438

[121] Wischnewski M Shirinpour S Alekseichuk I Lapid MI Nahas Z Lim KO . Real-time TMS-EEG for brain state-controlled research and precision treatment: a narrative review and guide. J Neural Eng. (2024) 21:061001. doi: 10.1088/1741-2552/ad8a8e, 39442548 PMC11528152

[122] Jannati A Oberman LM Rotenberg A Pascual-Leone A. Assessing the mechanisms of brain plasticity by transcranial magnetic stimulation. Neuropsychopharmacol. (2023) 48:191–208. doi: 10.1038/s41386-022-01453-8, 36198876 PMC9700722

[123] Kraus D Naros G Bauer R Khademi F Leão MT Ziemann U . Brain state-dependent transcranial magnetic closed-loop stimulation controlled by sensorimotor desynchronization induces robust increase of corticospinal excitability. Brain Stimul. (2016) 9:415–24. doi: 10.1016/j.brs.2016.02.007, 26970878

[124] Cioffi E Hutber A Molloy R Murden S Yurkewich A Kirton A . EEG-based sensorimotor neurofeedback for motor neurorehabilitation in children and adults: A scoping review. Clin Neurophysiol. (2024) 167:143–66. doi: 10.1016/j.clinph.2024.08.009, 39321571 PMC11845253

